# Unique epithelial proliferative transcriptomic signature in proton pump inhibitor–responsive pediatric eosinophilic esophagitis

**DOI:** 10.1172/jci.insight.178595

**Published:** 2025-10-08

**Authors:** Somdutta Chakraborty, Ankit Sharma, Sahiti Marella, Christian F. Rizza, Patrick A. O’Brien, Varsha Ganesan, Gila Idelman, Susie Min, Mayee Chen, Talaya McCright-Gill, Nancy Gonzalez, Alexandros D. Polydorides, Paul S. Foster, Simon P. Hogan, Mirna Chehade

**Affiliations:** 1 Mary H. Weiser Food Allergy Center,; 2 Molecular Pathology Graduate Program, Department of Pathology, and; 3 Department of Internal Medicine, Michigan Medicine, University of Michigan, Ann Arbor, Michigan, USA.; 4 Mount Sinai Center for Eosinophilic Disorders and; 5 Department of Pathology, Molecular and Cell-Based Medicine, Icahn School of Medicine at Mount Sinai, New York, New York, USA.; 6 Faculty of Medicine, Health and Human Sciences, Woolcock Institute of Medical Research, Macquarie University, New South Wales, Australia.

**Keywords:** Gastroenterology, Immunology, Inflammation, Allergy, Cellular immune response

## Abstract

Clinical trials have identified 2 distinct eosinophilic esophagitis (EoE) treatment phenotypes: those that show proton pump inhibitor (PPI) responsiveness (PPI-R) and those that show PPI unresponsiveness (PPI-UR). Comprehensive clinical, endoscopic, and RNA-Seq analyses of patients with EoE prior to and following PPI therapy have not previously been performed to our knowledge. We showed that clinical, endoscopic, and histologic evaluation of esophageal biopsies from pediatric PPI-R and PPI-UR individuals with EoE prior to PPI therapy (diagnosis) were indistinguishable. RNA-Seq analyses revealed common immune and inflammatory transcriptional signatures in both PPI-R EoE and PPI-UR EoE esophageal biopsy samples at diagnosis and distinct signatures enriched for processes related to neuropeptide signaling and cell cycle and division. PPI therapy induced histologic, endoscopic, and transcriptional remission in PPI-R EoE, but not in PPI-UR EoE. Persistent disease in PPI-UR EoE was associated with the presence of Th2 inflammatory and dedifferentiated esophageal epithelial transcriptomic signatures, while PPI-R EoE revealed genes enriched in cellular responses to LPS, host defense against viruses, and type I IFN signaling. In silico analyses identified common and unique EoE disease gene drivers in PPI-R and PPI-UR EoE. These studies indicate that the 2 EoE phenotypes have unique transcriptomic elements that underlie the molecular nature of PPI-R and PPI-UR EoE disease.

## Abstract 2

These studies show significant divergence in the PPI-R and PPI-UR EoE transcriptomic signature at diagnosis and identify key drivers of the EoE phenotype and PPI-unresponsiveness.

## Introduction

Eosinophilic esophagitis (EoE) is an emerging chronic inflammatory disease of the esophagus characterized by upper gastrointestinal symptoms, including dysphagia and esophageal food impaction (1, 2). Histopathological features of EoE include intraepithelial infiltration of eosinophils (eos) (≥15 eos/high-powered field [HPF]) and remodeling of the esophageal epithelium, including basal zone hyperplasia (BZH) and dilated intercellular spaces (DIS), which can lead to fibrostenotic sequelae of strictures and narrow-caliber esophagus (3, 4).

Both clinical observations and laboratory studies support the concept that EoE pathogenesis involves a food allergen–triggered CD4^+^ T helper type 2–predominant (Th2-predominant) immune response within the esophageal mucosa (5–9). Esophageal epithelial cell release of alarmin cytokines (e.g., thymic stromal lymphopoietin and IL-33) are thought to promote recruitment and expansion of CD4^+^ Th2 cells. These activated CD4^+^ Th2 cells produce key effector cytokines, including IL-5 and IL-13, that facilitate the recruitment and activation of the allergic effector cells, eos, and mast cells. Mast cell– and eos-derived mediators are thought to stimulate dysregulation of epithelial barrier regulatory and proliferative response genes within the esophageal epithelial compartment, leading to esophageal epithelial remodeling and fibrosis.

Prior to 2018, individuals who experienced signs and symptoms consistent with EoE underwent an 8- to 12-week treatment trial with a proton pump inhibitor (PPI) at a high dose, with approximately 50% individuals achieving clinicohistological remission (10). This treatment response pattern established the recognition of 2 distinct EoE treatment phenotypes: those demonstrating PPI responsiveness (PPI-R EoE) versus those showing PPI nonresponsiveness (PPI-UR EoE) (11, 12). Comparative retrospective studies examining these patient populations following PPI trial revealed no discernible differences in clinical presentation, endoscopic findings, or histological features (13–19). Furthermore, atopy status and transcriptomic molecular signature were largely similar between the 2 phenotypes (20, 21). However, it remains currently unclear whether transcriptional signature is indistinguishable prior to PPI trial. Identification of unique disease features to predict PPI treatment outcomes holds considerable clinical value, as it would (a) increase the chance of disease remission in a timely manner and avoid the need for additional invasive procedures to determine response (22) and (b) prevent disease progression and development of long-term complications in those patients unresponsive to PPI therapy. Collectively, this enables personalized precision medicine and improved patient outcomes.

Herein, we performed comprehensive clinical, endoscopic, histologic, and RNA-Seq analyses of PPI-R and PPI-UR EoE in pediatric individuals at diagnosis and following PPI therapy. We confirmed that PPI-R and PPI-UR individuals with EoE were clinically, endoscopically, and histologically indistinguishable at diagnosis. We observed a common prototypic EoE type 2 inflammatory transcriptomic gene profile in PPI-R and PPI-UR EoE in treatment-naive individuals at diagnosis; however, there was significant divergence in the transcriptional signature between PPI-R EoE and PPI-UR EoE following PPI treatment. Treatment-naive PPI-R EoE was enriched for genes associated with biological processes involved in LPS, defense response to viruses, and type I IFN signaling, whereas PPI-UR EoE possessed a unique B cell and neutrophil signature and a dominant DNA replication, cell cycle, and cell division signal. We showed that a PPI trial rectifies the prototypic EoE type 2 inflammatory transcriptomic gene signature in PPI-R EoE; however, the type 2 inflammatory and proliferative signature, while diminished, remains dominant in PPI-UR EoE. Strikingly, the recently identified secreted frizzled-related protein 1^+^ (SFRP1^+^) proinflammatory esophageal suprabasal (ESB) cell signature (*ALOX15*, *TNFAIP6*, *CCL26*, *POSTN*) was present in PPI-UR EoE and was unresponsive to PPI trial. These studies show significant divergence in PPI-R and PPI-UR EoE transcriptomic signature at diagnosis. They also demonstrate a potential role for the SFRP1^+^ ESB cell population as a contributor to the EoE phenotype and to PPI-UR.

## Results

### Characteristics of PPI-R and PPI-UR patients with EoE.

Ten pediatric patients with EoE (age, 10.4 ± 3.4 years; M/F, 6:4) underwent esophagogastroduodenoscopy (EGD) with biopsies and were diagnosed with EoE per consensus guidelines (23). Patients underwent PPI monotherapy (omeprazole or lansoprazole) for 8–12 weeks followed by repeat EGD with biopsies. PPI adherence for all patients was confirmed by the treating clinician prior to their repeat endoscopies. Of the *n* = 10 patients, 5 individuals demonstrated PPI-R, and 5 individuals were unresponsive to PPI monotherapy (Table 1, Table 2, and Supplemental Table 1; supplemental material available online with this article; https://doi.org/10.1172/jci.insight.178595DS1). We stratified the individuals into either PPI- R or PPI-UR groups and recruited participants matched for age, sex, and atopic status (age, 7.2 ± 3.5 years; M/F, 3:2) assigned as controls, who underwent EGD with biopsies. Participants acting as controls had 0 esophageal eos/HPF on biopsies and absent endoscopic findings inclusive of any EoE features on endoscopy, no esophageal or gastrointestinal pathology, and no other gastrointestinal diseases (Table 2 and Supplemental Table 1). Demographics and clinical, endoscopic, and histologic characteristics of the PPI-R and PPI-UR patients at diagnosis and following PPI monotherapy and the individuals acting as controls are described in Tables 1 and 2 and Supplemental Table 1. At diagnosis, the peak for esophageal eos/HPF (across all biopsy sites) was 94.2 ± 41.3 for the PPI-R group and 134.4 ± 49.6 for the PPI-UR group. Following PPI therapy, peak esophageal eos/HPF (across all biopsy sites) in the PPI-R group was 3.6 ± 2.6 and 125.6 ± 61.0 in the PPI-UR group. In the distal esophagus, where biopsies for RNA-Seq analyses were obtained, peak esophageal eos/HPF was 87.8 ± 46.9 for the PPI-R group and 132.2 ± 48.7 for the PPI-UR group at diagnosis. Following PPI therapy, peak distal esophageal eos/HPF in the PPI-R group was 3.0 ± 1.9 and 66.6 ± 46.0 in the PPI-UR group (Table 2). We observed statistically significant improvement in endoscopic (endoscopic reference score [EREFS]) and histologic (EoE histologic scoring system [EoE-HSS]) outcomes in PPI-R patients with EoE following PPI therapy (EREFS, total distal score, 3.4 ± 0.9 vs. 0.8 ± 0.8; EoE-HSS, distal grade score, 0.55 ± 0.14 vs. 0.12 ± 0.11; data represented as diagnosis vs. after PPI therapy, *P* < 0.01) (Table 2 and Supplemental Table 1). The PPI-R and PPI-UR groups had no statistically significant differences in mean age, M/F ratio, atopic status, and peak esophageal eos/HPF, EREFS, and EoE-HSS at diagnosis (Tables 1 and 2).

### RNA-Seq analyses at diagnosis.

**Figure 1:**
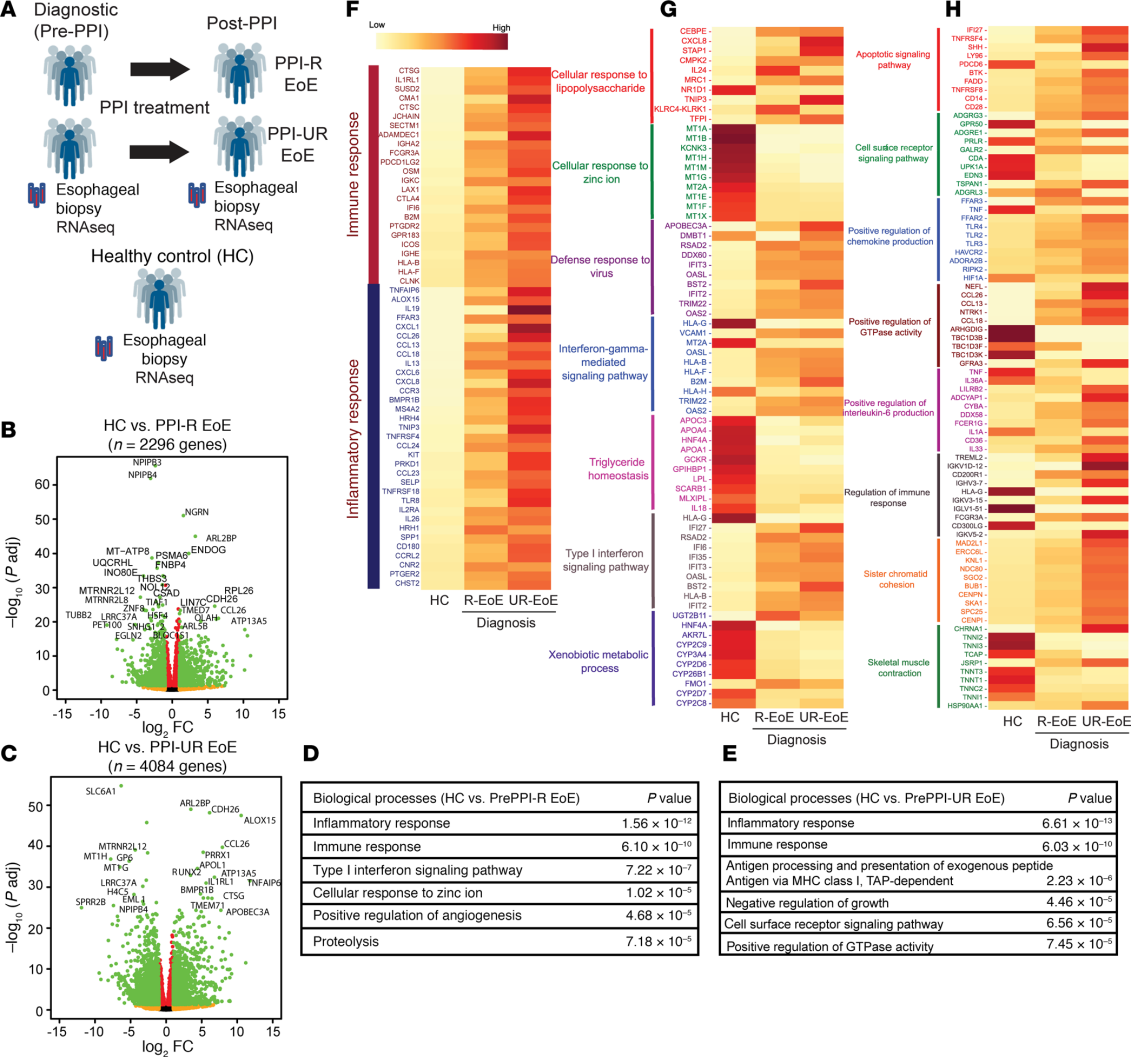
RNA-Seq analysis of PPI-R and PPI-UR patients with EoE at diagnosis. (**A**) Summary schematic of 25 esophageal biopsy samples (*n* = 5 PPI-R at diagnosis, *n* = 5 PPI-R following PPI therapy, *n* = 5 PPI-UR at diagnosis, *n* = 5 PPI-UR following PPI therapy, and *n* = 5 healthy controls). (**B** and **C**) Comparative gene expression of DEGs between treatment-naive (**B**) individuals acting as healthy controls and PPI-R patients with EoE (FDR < 0.05; log_2_FC > 1) and (**C**) individuals acting as healthy controls and PPI-UR patients with EoE (FDR < 0.05; log_2_FC > 1). (**D** and **E**) Comparison of enriched biological processes and pathways in (**D**) individuals acting as healthy controls and PPI-R patients with EoE and (**E**) individuals acting as healthy controls and PPI-UR patients with EoE. (**F**) Heatmap of enriched genes associated with the immune and inflammatory response in individuals acting as healthy controls and PPI-R and PPI-UR patients with EoE at diagnosis. (**G** and **H**) Heatmap of enriched pathways (upregulated and downregulated) in individuals acting as healthy controls and PPI-R and PPI-UR patients with EoE at diagnosis.

A total of 25 esophageal biopsy samples from *n* = 5 PPI-R patients with EoE (at diagnosis [before PPI] and following 8- to 12-week PPI therapy), *n* = 5 PPI-UR patients with EoE (at diagnosis [before PPI] and following 8- to 12-week PPI therapy), and 5 healthy individuals acting as controls were collected and analyzed by RNA-Seq (Figure 1A). Data processing, normalization, and quality control resulted in *n* = 43,186 transcripts with at least 1 raw read in any 1 sample of any group that mapped to >98% human reference genome Grch38. We observed an average of 34,122 raw read counts in the control samples, with 27,634 (82.48%) common genes expressed in each sample; an average of 21,446 raw read counts with 17,768 (82.8%) common genes expressed among PPI-R individuals; and an average of 23,521 raw read counts, with 18,343 (72.8%) common genes expressed among PPI-UR individuals.

We performed comparative gene expression analysis between individuals acting as controls and PPI-R and PPI-UR individuals with EoE at diagnosis using pairwise comparison cutoff criteria of FDR < 0.05; log_2_fold change (log_2_FC) > 1. We identified a total of 2,296 (1,022 upregulated and 1,274 downregulated) differentially expressed genes (DEGs) between healthy individuals acting as controls and PPI-R individuals with EoE and 4,084 (1,929 upregulated and 2155 downregulated) between individuals acting as controls and PPI-UR individuals with EoE (Figure 1, B and C, and Supplemental Table 2, A and B). DAVID pathway analyses revealed common dysregulation of inflammatory response and immune response pathways, in particular dysregulation of pro–type 2 genes previously identified as part of the EoE transcriptome (*CCL26*, *CCL24*, *TNAIP6*, *ALOX15*, *FFAR3*, *IL13*) as well as additional genes associated with chemotaxis (*CCL13*, *CCL18*, *CCL23*, *CXCL1*, *CXCL6*, and *CXCL8*), CD4^+^ type 2 immune genes (*TNFRSF4*, *PTGER2*), and IL-20 cytokine family members (*IL19* and *IL26*) (Figure 1, D–F; Supplemental Table 3, A and B; and Supplemental Table 4A). Notably, the immune and inflammatory response genes were markedly more dysregulated (higher expression of corresponding genes) in PPI-UR individuals with EoE than in PPI-R individuals with EoE at diagnosis (Figure 1F and Supplemental Table 4A). Examination of the DEGs and most significantly enriched pathways within PPI-R EoE and PPI-UR EoE groups at diagnosis revealed significant upregulation of pathways involved in “cellular response to LPS,” “defense response to virus,” “IFN-gamma-mediated signaling,” and “type I IFN signaling pathways,” and downregulation of “cellular response to zinc ion,” “triglyceride homeostasis,” and “xenobiotic metabolic process*”* pathways in PPI-R EoE (Figure 1, D and G; Supplemental Table 3A; and Supplemental Table 4B). In contrast, the most enriched dysregulated pathways observed in the PPI-UR EoE group at diagnosis were associated with “apoptotic signaling pathways,” “positive regulation of chemokine production GTPase activity,” “IL6 production,” and “sister chromatid cohesion” (Figure 1, E and H; Supplemental Table 3B; and Supplemental Table 4C). The genes associated with the enriched pathways (upregulated and downregulated) were significantly more dysregulated in the PPI-UR group than the PPI-R group (Figure 1, F–H). To validate our RNA-Seq analyses, we compared our RNA-Seq data with previously reported transcriptomic profiling analyses performed on adult and pediatric EoE esophageal biopsies (GSE58640, GSE197702) (24). We showed that there was significant overlap in our PPI-R and PPI-UR EoE groups at diagnosis with that described in adult and pediatric EoE esophageal biopsies (Supplemental Figure 1A). Furthermore, when comparing the FC of DEGs between studies, we found a significant correlation in expression of DEGs within the external datasets and PPI-R and PPI-UR EoE at diagnosis (Supplemental Figure 1B). Collectively, these studies suggest common and unique dysregulated genes and pathways between PPI-R EoE and PPI-UR individuals with EoE at diagnosis and that there are conserved molecular signatures in EoE regardless of age group or PPI-R.

**Figure 2:**
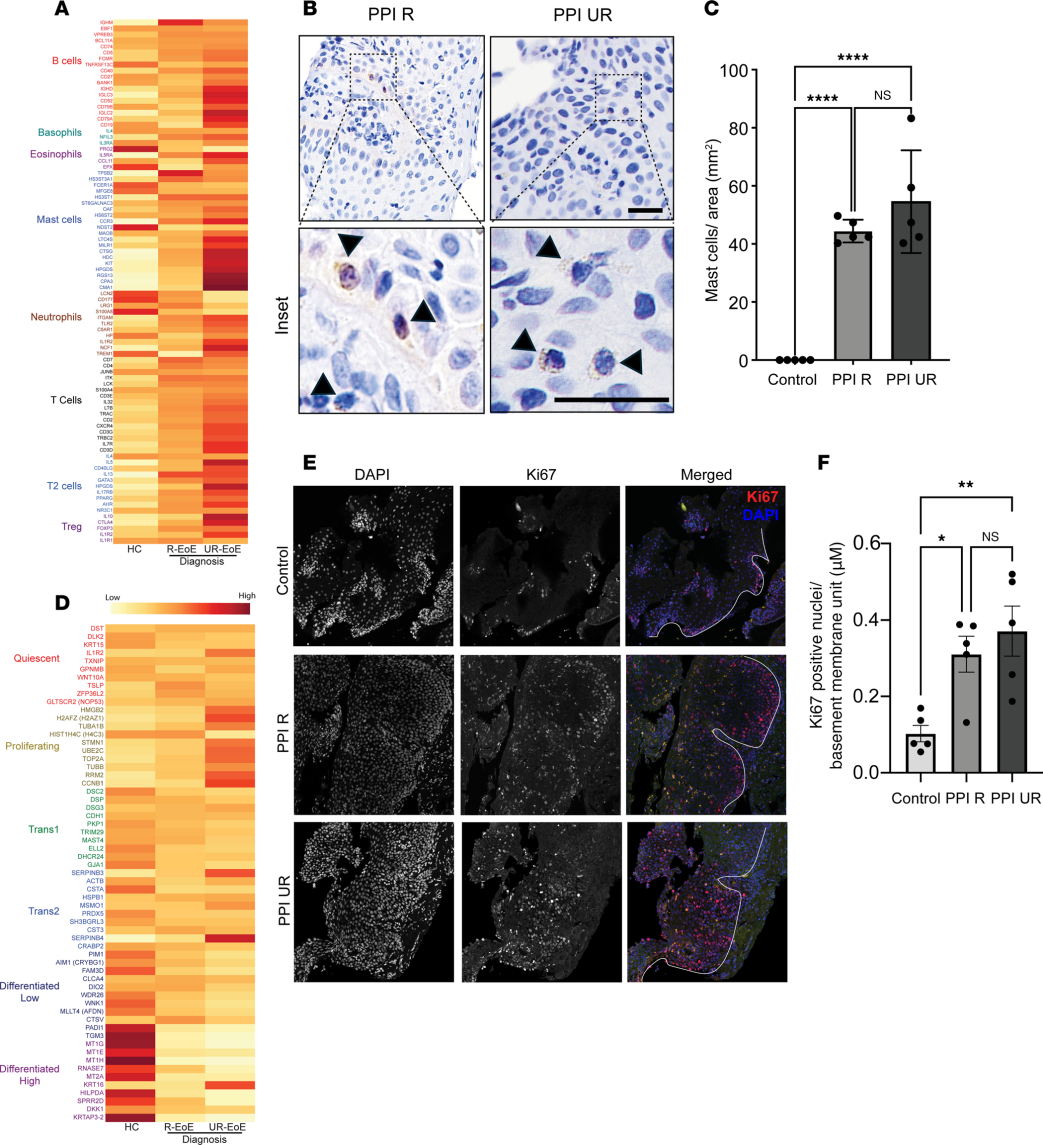
Hematopoietic and esophageal epithelial transcriptomes in PPI-R and PPI-UR EoE at diagnosis. (**A**) Heatmap of the gene expression profile of hematopoietic cell–specific markers in individuals acting as healthy controls and PPI-R and PPI-UR patients with EoE at diagnosis. (**B**) IHC-stained esophageal biopsies from PPI-R and PPI-UR patients showing tryptase^+^ mast cells (inset). (**C**) Quantifications of mast cells per unit area of biopsied tissue in individuals acting as healthy controls and PPI-R and PPI-UR patients (*n* = 5 per group). (**D**) Heatmap of the gene expression profile of esophageal epithelial cell type–specific markers in individuals acting as healthy controls and PPI-R and PPI-UR patients with EoE at diagnosis. (**E**) Confocal images of esophageal biopsies of individuals acting as healthy controls and PPI-R and PPI-UR patients, showing nuclei (DAPI) and proliferating cells (Ki67). Basement membrane is traced in white. (**F**) Quantifications of proliferating cells along the basement membrane in individuals acting as healthy controls and PPI-R and PPI-UR patients (*n* = 5 per group). (**C** and **F**) 1-way ANOVA was used to determine significance between the groups; *P < 0.05, **P < 0.01, ****P < 0.0001.

We next examined the involvement of the hematopoietic cell compartment in PPI-R and PPI-UR EoE at diagnosis. To do this, we mapped hematopoietic cell–specific gene markers identified using the PangloDB database (http://panglaodb.se) onto the normalized gene expression profile of PPI-R and PPI-UR EoE transcriptome at diagnosis. We identified high expression of mast cell (*CMA1*, *CPA3*, *CKIT*, *CTSG*, and *RGS13*), eos (*EPX* and *IL5RA*), and CD4^+^ Th2 cell (*AHR*, *GATA3*, *IL13*, *IL17RB*) signature genes both in PPI-R EoE and PPI-UR EoE at diagnosis (Figure 2A and Supplemental Table 5, A–C). Overall, the type 2 immune cell gene signature was enhanced in the treatment-naive PPI-UR EoE group compared with PPI-R EoE group (Figure 2A). Notably, we observed enrichment of a neutrophil (*ITGAM*, *LCN2*, *NCF1*, *S100A8*, and *TLR2*) and B cell signature (*IGHD, IGLC3, CD52, IGLC2, CD79A*, and *CD19*) in the PPI-UR EoE group that was not observed in PPI-R EoE at diagnosis (Figure 2A and Supplemental Table 5, A–C). To validate these observations, we performed immunofluorescence and histochemistry analyses on esophageal biopsies from PPI-R and PPI-UR individuals with EoE at diagnosis staining for mast cells (anti-tryptase) and evidence of esophageal epithelial proliferation (anti-Ki67). We observed that Tryptase^+^ mast cell numbers were significantly increased in both PPI-R and PPI-UR EoE compared with controls at diagnosis (Figure 2, B and C).

Utilizing the gene signatures derived from single-cell RNA-Seq (scRNA-Seq) analyses of esophageal epithelial populations (GSE201153) (25), we examined esophageal epithelial cell population gene expression in PPI-R and PPI-UR EoE at diagnosis. We identified the presence of quiescent and proliferating basal cells, Trans1 and Trans2 suprabasal cells, and low and high differentiated squamous epithelial cell transcriptomes in control individuals (Figure 2D and Supplemental Table 6). Consistent with a predominant mature squamous esophageal epithelium, differentiated squamous epithelial cell genes were highly enriched as compared with suprabasal and basal cell genes in esophageal biopsy samples from control individuals (Figure 2D and Supplemental Table 6). Notably, we observed dedifferentiation of the esophageal epithelial layer in both PPI-R and PPI-UR EoE at diagnosis as indicated by decreased expression of low differentiated genes (*WDR26*, *WNK1*, *PADI1*, and *TGM3*) and high differentiated (*MT1G*, *MT1E*, *MT1H*, *HLPDA*, and *KRTAP3*) esophageal epithelial cell genes. Furthermore, we observed enriched expression of proliferating epithelial genes (*HMGB2*, *H2AFZ*, *TUBA1B*, *STMN1*, *UBE2C*, *TOP2A*, *RRM2*, and *CCNB1*) in PPI-UR EoE at diagnosis, suggesting increased esophageal epithelial proliferation. Intriguingly, PPI-UR EoE at diagnosis had greater expression of proliferating epithelial genes (*HMGB2*, *H2AFZ*, *TUBA1B*, *STMN1*, *UBE2C*, *TOP2A*, *RRM2*, and *CCNB1*) as compared with PPI-R EoE (Figure 2D and Supplemental Table 6). Ki-67 staining of distal esophageal biopsies of pediatric individuals revealed that the level of epithelial proliferation was significantly higher in PPI-R and PPI-UR patients with EoE compared with that in individuals acting as controls at diagnosis (Figure 2, E and F). While the number of Ki-67^+^ esophageal epithelial cells was higher in the PPI-UR EoE group compared with PPI-R EoE group, the level of epithelial proliferation was not significantly different between groups. Collectively, these data support common CD4^+^ Th2 cell, eos and mast cell inflammatory signature, and dedifferentiation of the esophageal epithelium in PPI-R and PPI-UR EoE at diagnosis and that PPI-UR EoE at diagnosis was associated with enrichment of esophageal proliferative gene signature.

### Relationship between gene expression and endoscopic severity.

We next examined if any DEGs correlated with endoscopic parameters (EREFS score) in PPI-R and PPI-UR EoE at diagnosis. We show that the EREFS score of the distal esophagus was significantly elevated in PPI-R and PPI-UR EoE at diagnosis and that PPI therapy significantly reduced the EREFS score in PPI-R EoE but not in PPI-UR EoE (Table 2 and Supplemental Figure 2A). Correlation analyses revealed a positive correlation between distal esophageal histologic parameters (peak eos/HPF, BZH, DIS, and grade total score) and EREFS total distal score (peak count *P* = 0.002; *r* = 0.72, DIS *P* = 9.51 × 10^–6^; *r* = 0.89, BZH *P* = 6.87 × 10^–7^; *r* = 0.92 and total grade score *P* = 1.93 × 10^–5^; *r* = 0.88) (Supplemental Figure 2B and Supplemental Table 7). Correlation analysis between endoscopic parameter (total distal score) and DEGs in PPI-R and PPI-UR EoE at diagnosis identified that 2,064 of 2,296 genes correlated with the total distal EREFS score in PPI-R EoE and 3,527 of 4,084 genes correlated with total distal EREFS score in PPI-UR EoE (Supplemental Figure 2C and Supplemental Table 7). Gene ontology (GO) analysis analyses identified enrichment of genes associated with biological processes, including “inflammatory response” and “detoxification of copper ion”, which correlated with EREFS score in both PPI-R and PPI-UR EoE groups. However, biological processes such as “type I IFN signaling pathway,” “defense response to virus,” and “apoptotic signaling pathway,” uniquely correlated with EREFS score in PPI-R EoE and “cornification,” “chromosome segregation,” and “mitotic spindle organization” were specific for PPI-UR EoE (Supplemental Figure 2D).

### Relationship between gene expression and histological characteristics.

**Figure 3:**
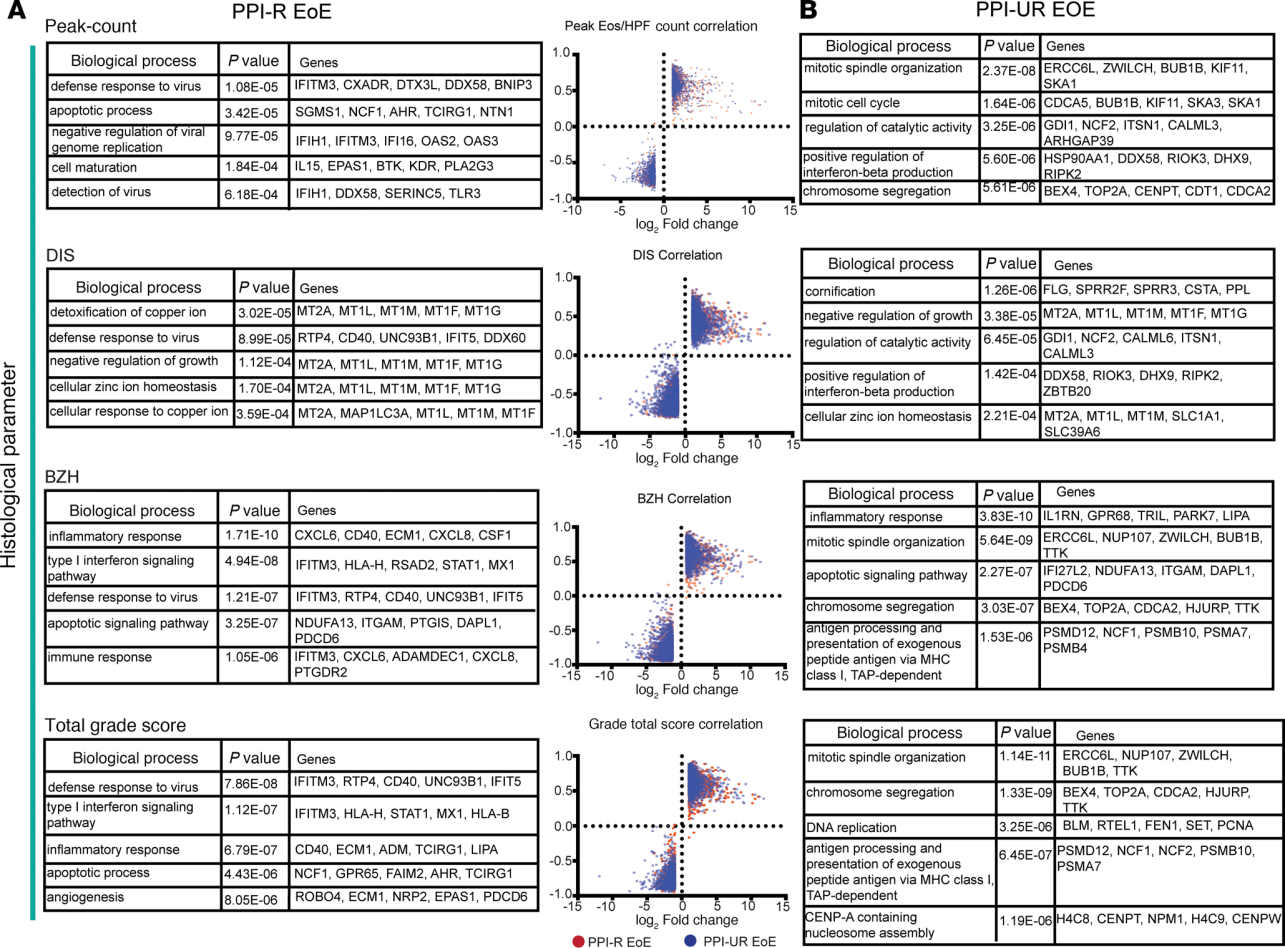
Relationship between gene expression and endoscopic severity in PPI-R and PPI-UR EoE at diagnosis. (**A**) DEGs and associated pathways that correlated with peak eosinophil count, DIS, BZH, and total histological score in PPI-R patients with EoE at diagnosis. Histograms represent a flatten matrix correlation analyses of DEGs in PPI R- and PPI-UR EoE at diagnosis with the histological parameters of the distal esophagus determined using the EoE-HSS score criteria. Red symbol represents PPI-R EoE and blue symbol PPI-UR EoE. (**B**) DEGs and associated pathways that correlated with peak eosinophil count, DIS, BZH, and total histological score in PPI-UR EoE at diagnosis.

We next examined the relationship between DEGs in PPI-R EoE and PPI-UR EoE at diagnosis and histologic alterations, including eos/HPF, BZH, and DIS. To do this we performed a flatten matrix correlation analyses of 2,296 and 4,084 DEGs in PPI R- and PPI-UR EoE at diagnosis with the histological parameters of the distal esophagus determined using the EoE-HSS score criteria (26). We identified that 681 of 2,296 DEGs in PPI-R individuals with EoE correlated with peak eos/HPF count; 1,421 of 2,296 DEGs correlated with DIS; 1,745 of 2,296 DEGs correlated with BZH; and 1,332 of 2,296 DEGs correlated with the distal EoE-HSS total grade score. To get insight into the biological processes that contribute to the histologic alterations, eos/HPF, BZH, DIS, and distal EoE-HSS grade total score, we performed GO analyses on the DEGs that correlated with the individual histologic characteristics (Figure 3). We show that the biological processes “defense response to virus” and “apoptotic process” were enriched with peak eos/HPF count, BZH, and total grade score. Furthermore, the biological processes “inflammatory response” and “type I IFN signaling pathway” were enriched in BZH and total grade score, suggesting that these biological processes contribute to these histologic characteristics (Figure 3 and Supplemental Tables 8 and 9). Intriguingly, DEGs that correlated with DIS in PPI-R EoE were enriched for the biological processes associated with “detoxification of copper ion,” “defense response to virus,” “negative regulation of growth,” “cellular zinc ion homeostasis,” and “cellular response to copper ion,” suggesting that processes that regulate DIS are distinct from those associated with eos/HPF and BZH (Figure 3A and Supplemental Table 9, A–D). The DEGs that dominated the signature were the metallothionein (MT) genes (*MT2A*, *MT1L*, *MT1M*, *MT1F*, *MT1G*), which are low-molecular-weight metal binding proteins that play an important role in regulating metal homeostasis and controlling physiological heavy metal toxicity, DNA damage, and oxidative stress (27). In PPI-UR EoE, of the 4,084 DEGs, 1,544 DEGs correlated with distal peak eos count; 2,201 DEGs correlated with DIS; 3,113 correlated with BZH; and 2,618 correlated with distal grade total score (Supplemental Table 9, E–H). In UR EoE, there was overlap in the enriched biological processes, such as “mitotic spindle organization” and “chromosome segregation,” that correlated with peak eos/HPF count, BZH, and total grade score (Figure 3B). Furthermore, there were common biological processes, including “mitotic spindle organization,” “chromosome segregation,” and “antigen processing and presentation of exogenous peptide antigen via MHC class I, TAP-dependent,” that correlated between BZH and total grade Score. Notably, the top enriched biological processes that correlated with the histologic characteristics, peak eos/HPF count, BZH, and total grade score in PPI-R EoE and PPI-UR EoE at diagnosis were different. The biologic processes “defense response to virus,” “inflammatory response,” and “type I IFN signaling pathway” were dominant in PPI-R EoE whereas “mitotic spindle organization” and “chromosome segregation” was most enriched in PPI-UR EoE (Figure 3 and Supplemental Table 9, A–H). The top biologic processes that correlated with DIS in UR EoE had the least overlap with the other histologic characteristics, peak eos/ HPF count, BZH and total grade score. Interestingly, the most enriched biologic processes in PPI-UR EoE were like those observed in PPI-R EoE, including “negative regulation of growth” and “cellular zinc ion homeostasis,” that possessed a strong MT gene signature and supported a role for these processes in DIS in both PPI-R and PPI-UR EoE (Figure 3 and Supplemental Table 9, A–H).

### Common and unique transcriptome network and pathways between PPI-R EoE and PPI-UR EoE at diagnosis.

**Figure 4:**
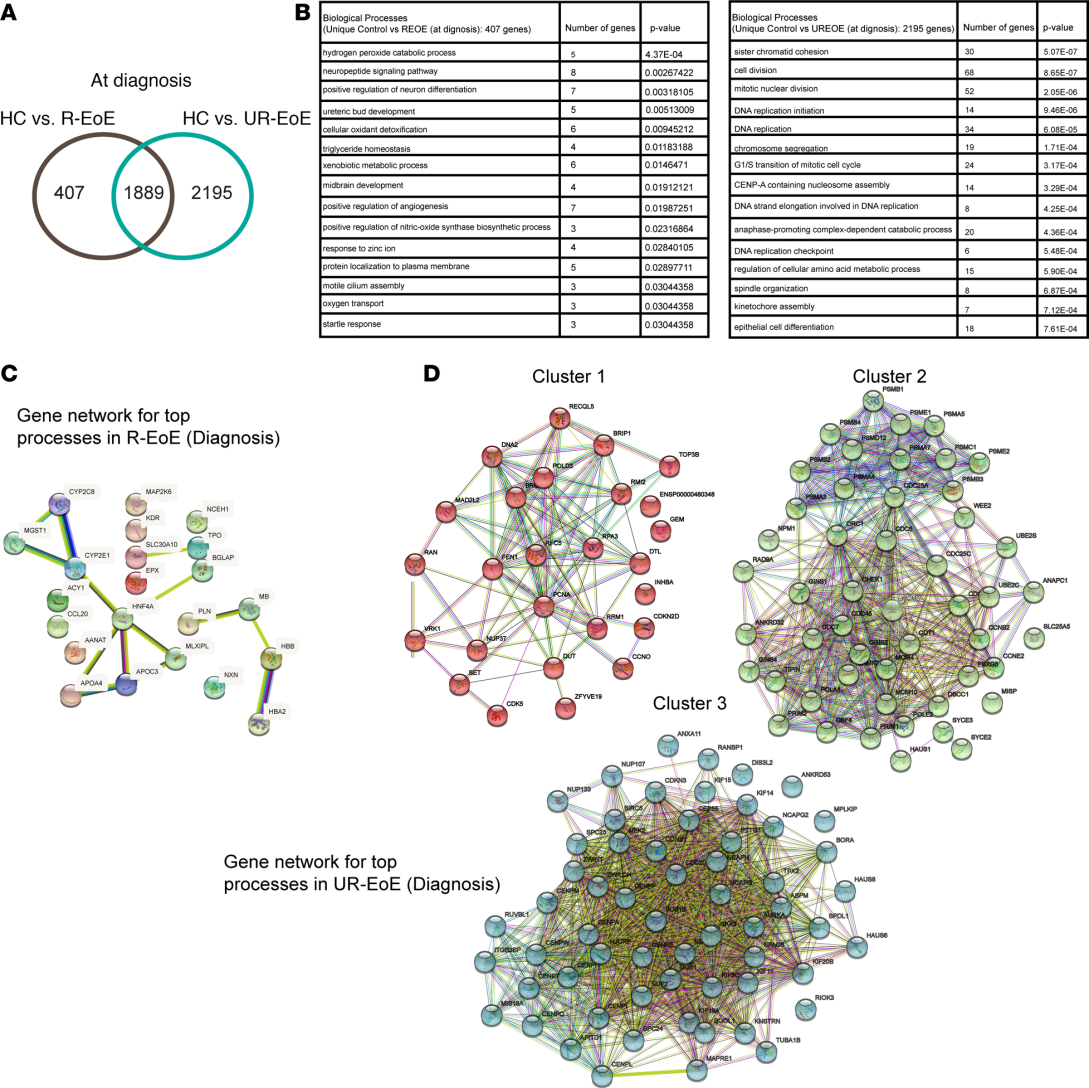
Common and unique transcriptome network and pathways between PPI-R and PPI-UR EoE at diagnosis. (**A**) Venn diagram depicting the common and unique DEGs between individuals acting as healthy controls and PPI-R patients and individuals acting as healthy controls and PPI-UR patients at diagnosis. (**B**) Gene ontology enrichment analysis of unique DEGs in PPI-R and PPI-UR patients at diagnosis. (**C**) K-means clustering interaction network on the DEGs associated with top enriched biological processes in PPI-R EoE at diagnosis and (**D**) K-means clustering interaction networks (clusters 1–3) on the DEGs associated with top enriched biological processes in PPI-UR EoE at diagnosis.

To determine the common and unique expressed genes between PPI-R EoE and PPI-UR EoE at diagnosis we mapped the DEGs at diagnosis in PPI-R EoE onto the PPI-UR EoE (Supplemental Table 10). We identified a total of 1,889 common DEGs between PPI-R EoE and PPI-UR EoE at diagnosis and 407 unique DEGs in PPI-R EoE and 2,195 DEGs in PPI-UR EoE (Figure 4A and Supplemental Table 10, A–C). GO analysis of unique DEGs in PPI-R EoE identified enrichment of pathways associated with “hydrogen peroxide catabolic process,” “cellular oxidant detoxification,” “triglyceride homeostasis,” “xenobiotic metabolic process,” “positive regulation of nitric-oxide synthase biosynthetic process,” and “response to zinc ion and oxygen transport” (Figure 4B and Supplemental Table 10D). K-mean clustering interaction network analysis performed on the DEGs identified in the GO analysis revealed strong network interactions between genes, including *CYP2E1*, *HNF4A*, *APOA3*, *APOC4* and *MLXIPL*, and *BGLAP*. Other DEGs, including *PLN*, *MB*, *HBB*, *HBA2*, *SLC30A10*, and *TPO*, demonstrated low level interactivity (Figure 4C). The 2,195 unique DEGs in PPI-UR EoE were enriched for pathways involved in “sister chromatid cohesion,” “cell division,” “mitotic nuclear division,” “DNA replication initiation,” “DNA replication,” “chromosome segregation,” “CENP-A containing nucleosome assembly,” “DNA strand elongation involved in DNA replication,” “anaphase-promoting complex- dependent catabolic process,” and “DNA replication checkpoint” (Figure 4B and Supplemental Table 10E). K-mean clustering network analysis of DEGs in PPI-UR EoE identified 3 major interaction clusters associated with cell cycle, cell division, and proliferation (Figure 4D). The cluster 1 protein network consisted of genes, including *RPA3*, *BRIP1*, *POLD3*, *RRM1*, *FEN1*, *VRK1*, *RPA3*, *MAD2L2*, *INHBA*, *BRIP1*, *RPA3*, *MAD2L2*, *BRIP1*, *POLD3*, and *FEN1*, that are involved in “DNA replication cell cycle,” “cell processes,” and “mitotic cell cycle DNA metabolic processes” (Supplemental Table 10F). Cluster 2 consisted of genes, including *PSMA4*, *CDC6*, *PSMA3*, *PSME2*, *FBXO5*, *MISP*, *POLE2*, and *TIPIN* that are involved in “regulation of cell cycle phase transition,” “mitotic cell cycle process,” “regulation of cell cycle process,” and “regulation of mitotic cell cycle and cell cycle process.” Cluster 3 consisted of genes, including *AURKA*, *NCAPH*, *KNSTRN*, *NCAPG*, *HAUS8*, *NCAPH*, *KNSTRN*, *NCAPG*, *CCNB1*, and *NDC80*, involved in “chromosome segregation,” “cell division,” “cell cycle,” “mitotic cell cycle,” “cell cycle process,” and “chromosome segregation” (Figure 4D and Supplemental Table 10, G and H). Collectively, these studies reveal a highly enriched esophageal epithelial proliferative transcriptome unique to PPI-UR EoE at diagnosis.

### PPI effect on the transcriptome profiles between different disease groups.

**Figure 5:**
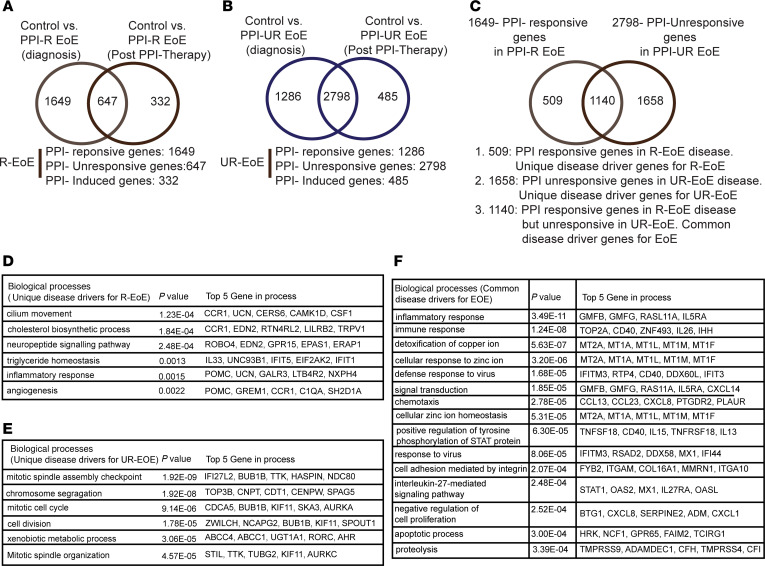
The effect of PPI treatment on the transcriptome profiles between PPI-R and PPI-UR EoE. (**A** and **B**) Venn diagram of the DEGs at diagnosis versus after PPI treatment in PPI-R (**A**) and PPI-UR (**B**) EoE (*P* < 0.05, FC > 2) (**C**) Venn diagram of disease-associated DEGs in PPI-R EoE and disease-associated DEGs in PPI-UR EoE. (**D**) Biological processes associated with disease-associated DEGs in PPI-R EoE, and (**E**) biological processes associated with disease-associated DEGs in PPI-UR EoE. (**F**) Biological processes that are common disease drivers associated with EoE.

Upon diagnosis, individuals with EoE underwent 8- to 12-week PPI therapy, and follow-up endoscopic, histologic, and transcriptomic analyses were performed. In PPI-R individuals, we observed that PPI therapy induced disease remission characterized by a significant reduction in histologic (eos/HPF, BZH, and DIS) and endoscopic involvement (EFRES) (Table 2 and Supplemental Table 1). Transcriptomic analyses revealed that PPI therapy in PPI-R individuals with EoE led to a significant reduction in DEGs compared with that in individuals acting as controls (2,296 vs. 979 DEGs, *P*_adj_ < 0.05, FC > 2; Figure 5 and Supplemental Table 11). Mapping the DEGs in PPI-R individuals with EoE at diagnosis onto the DEGs following PPI therapy revealed 1,649 genes that are PPI responsive, 647 that are PPI unresponsive, and 332 that are PPI-induced genes in PPI-R EoE (*P*_adj_ < 0.05, FC > 2, Figure 5A and Supplemental Table 11A). Notably, differentially expressed hematopoietic cell–specific genes for eos, mast cells, and CD4^+^ Th2 cells returned to levels comparable to those of control individuals (Supplemental Figure 3A). Furthermore, the dedifferentiated esophageal epithelial transcriptomic signature observed at diagnosis resembled that of control individuals with enrichment of a mature differentiated esophageal epithelial signature (Supplemental Figure 3B). Consistent with the observed reduction in inflammatory and histological alterations, the genes that were elevated in PPI-R EoE at diagnosis associated with the biological processes “immune response” and “inflammatory response” resembled that of control individuals (Supplemental Figure 4A). Furthermore, the DEGs upregulated at diagnosis associated with the biological processes “cellular response to LPS,” “defense response to virus,” “IFN-gamma-mediated signaling,” and “type I IFN signaling pathways,” and downregulated DEGs involved in “cellular response to zinc ion,” “triglyceride homeostasis,” and “xenobiotic metabolic process pathways” also returned to levels comparable to control individuals (Supplemental Figure 4B and Supplemental Table 11, A and C). Notably, we identified *n* = 647 DEGs that were PPI unresponsive in PPI-R individuals with EoE (Figure 5A and Supplemental Table 11D). The DEGs that were PPI unresponsive were enriched for genes involved in the biological processes, including “arachidonic acid metabolic process” (*GPX1*, *CYP2D7*, *CYP2D6*, *PLA2G4B*, *ALOX15*, *ALOX12*, *CYP4F12*), “Rap1/Ras signaling pathway” (*PDGFRB*, *RASA4B*, *CALML6*, *PLA2G4B*, *FLT4*, *CALML4*, *PLA2G6*, *VEGFA*, *FGF17*, *RASA4*, *JMJD7*, *PLA2G4B*, *PDGFD*, *GNB3*, *LAT*, *FGF22*), and “neutrophil extracellular trap formation” (*H4C8*, *H2BC9*, *H2AW*, *HDAC10*, *ITGA2B*, *H2AC19*, *CYBA*, *AGER*, *SEL*, *H2AC20*, *CTSG*, *H4C4*, *H4C5*) (Supplemental Table 11, A and D).

Finally, there were *n* = 332 DEGs induced by PPI therapy in PPI-R individuals with EoE that were enriched for “gated channel activity” (*KCND1*, *GRIN3B*, *TRPA1*, *KCNH4*, *CACNA1C*, *CACNG8*, *KCNE4*, *ASIC3*, *CNGB3*, *CACNB2*, *CACNA1H*, *ZACN*, *TMC3*, *HCN3*, *LRRC26*, *GRIK3*, *CNGA4*, *GABRR2*) (Figure 5A and Supplemental Table 11, A and E).

In PPI-UR individuals with EoE, 8- to 12-week PPI therapy did not demonstrate any statistically significant reduction in eos/HPF (peak eos/HPF, 87.80 ± 46.9 vs. 132.2 ± 48.7, at diagnosis vs. following PPI trial; *n* = 5; mean ± SD) or reduction in histologic or endoscopic disease involvement (Table 2, Supplemental Table 1, and Supplemental Figure 1). While PPI trial in PPI-UR individuals with EoE did not induce disease remission (Table 2), transcriptomic analyses revealed that PPI trial reduced the number of DEGs when compared with individuals acting as controls (4,084 vs. 3283 DEGs, *P* < 0.05, FC > 2, Figure 5B). Mapping the DEGs in PPI-UR individuals with EoE at diagnosis onto the DEGs following PPI therapy revealed 1,286 genes that are PPI responsive, 2,798 that are PPI unresponsive, and 485 that are PPI-induced genes in PPI-UR EoE (*P* < 0.05, FC > 2, Figure 5B and Supplemental Table 11B). Consistent with the persistent inflammation and esophageal epithelial remodeling, the differentially expressed hematopoietic cell–specific genes for eos, mast cells and CD4^+^ Th2 cells remained significantly dysregulated, particularly the mast cell DEG signature (Supplemental Figure 3A). Furthermore, the dedifferentiated esophageal epithelial transcriptomic signature persisted in PPI-UR individuals with EoE following PPI therapy (Supplemental Figure 3B and Supplemental Table 11, B, F, and G). The level of expression of DEGs in PPI-UR EoE at diagnosis associated with the biological processes “immune response” and “inflammatory response” was reduced following PPI therapy but, in comparison to PPI-UR EoE at diagnosis, remained differentially expressed as compared with that of individuals acting as controls (Supplemental Figure 4A). Intriguingly, the common genes associated with the enriched biological processes in PPI-R EoE, such as “cellular response to LPS,” “defense response to virus,” and “type I IFN pathway,” and the genes and biological pathways specifically enriched in PPI-UR EoE at diagnosis, including “positive regulation of interleukin-6 production” and “sister chromatid cohesion,” remained differentially expressed following PPI therapy (Supplemental Figure 4, B and C). GO analysis revealed that the 1,286 DEGs that were PPI responsive in PPI-UR EoE were enriched for genes involved in “B cell receptor signaling pathway,” “immunoglobulin receptor binding,” “immunoglobulin complex,” and “complement activation, classical pathway” (Supplemental Table 11G). Paired analyses comparing gene expression between PPI-UR EoE at diagnosis and following PPI therapy identified no DEGs at a cutoff threshold of *P*_adj_ < 0.05, FC > 2.

### Disease-specific gene signature in PPI-R and PPI-UR EoE.

Our combined clinical and transcriptomic analyses suggest that the *n* = 1,649 DEGs that were PPI responsive in PPI-R EoE and the *n* = 2,798 DEGs that were PPI unresponsive in PPI-UR EoE are disease-specific gene drivers. To determine whether the transcriptomic programs that drive PPI-R and PPI-UR EoE are similar, we mapped the *n* = 1,649 disease-associated DEGs in PPI-R EoE onto the *n* = 2,798 disease- associated DEGs in PPI-UR EoE (Figure 5C and Supplemental Table 12, A–D). We identified 509 genes that were PPI-R EoE–specific gene drivers that were enriched for the biological processes involved in “cilium movement” (*CCR1*, *UCN*, *CERS6*, *CAMK1D*, *CSF1*), “cholesterol biosynthetic process” (*CCR1*, *EDN2*, *RTN4RL2*, *LILRB2*, *TRPV1*), “neuropeptide signaling pathway” (*ROBO4*, *EDN2*, *GPR15*, *EPAS1*, *ERAP1*), “triglyceride homeostasis” (*IL33*, *UNC93B1*, *IFIT5*, *EIF2AK2*, *IFIT1*), “inflammatory response” (*POMC*, *UCN*, *GALR3*, *LTB3R2*, *NXPH4*), and “angiogenesis” *(POMC*, *GREM1*, *CCR1*, *C1QA*, *SH2D1A*) (Figure 5, C and D, and Supplemental Table 12C). There were 1,658 genes that were PPI-UR EoE–specific gene drivers, including genes enriched for “mitotic spindle assembly checkpoint” (*ZWILCH*, *BUB1B*, *TTK*, *HASPIN*, *NDC80*), “chromosome segregation” (*TOP3B*, *CENPT*, *CDT1*, *CENPW*, *SPAG5*), “mitotic cell cycle” (*CDCA5*, *BUB1B*, *KIF11*, *SKA3*, *AURKA*), “cell division” (*ZWILCH*, *NCAPG2*, *BUB1B*, *KIF11*, *SPOUT1*), “xenobiotic metabolic process” (*ABCC4*, *ABCC1*, *UGT1A1*, *RORC*, *AHR*), and “mitotic spindle organization” (*STIL*, *TTK*, *TUBG2*, *KIF11*, *AURKC*) (Figure 5, C and E, and Supplemental Table 12D). Importantly, these analyses also revealed *n* = 1,140 genes that we defined as common EoE disease gene drivers, which included “inflammatory response” (*GMFB*, *GMFG*, *RASL11A*, *IL5RA*), “immune response” (*TPO2A*, *CD40*, *ZNF493*, *IL26*, *IHH*), “defense response to virus” (*STAT1*, *STING1*, *IRF1*, *TLR8*, *TLR3*, *TLR2*), “detoxification of copper ion” (*MT2A*, *MT1A*, *MT1L*, *MT1M*, *MT1F*) *“*cellular zinc ion homeostasis” (*MT2A*, *MT1A*, *MT1L*, *MT1M*, *MT1F*, *MT1G*), *“*defense response to virus” (*IFITM3*, *RTP4*, *CD40*, *DDX60L*, *IFIT3*), and *“*chemotaxis” (*CCL13*, *CCL23*, *CXCL8*, *PTGDR2*, *PLAUR*) (Figure 5F and Supplemental Table 12B) genes. Collectively, these studies indicate the presence of common and unique transcriptomic elements that underlie PPI-R and PPI-UR EoE disease phenotypes.

## Discussion

Previous transcriptomic analyses of esophageal biopsy samples from individuals with EoE have identified an EoE transcriptomic signature that is enriched for genes that regulate several type 2 inflammatory processes (*CCL26*, *TNAIP6*, *ALOX15*, *IL13*). The original microarray study revealed an EoE transcriptome that consisted of 574 genes that differentiate patients with EoE from healthy individuals acting as controls and patients with chronic esophagitis (28). A subsequent RNA-Seq analysis expanded the EoE transcriptome and identified 1,607 dysregulated genes that distinguished patients with EoE from healthy control individuals (29). However, these studies were complicated by the heterogenous population of PPI-R and PPI-UR individuals within the EoE cohorts (28, 29). In 2015, a study utilizing the EoE diagnostic panel (EDP) panel revealed elevated elements of the EoE transcriptomic signature (*TNAIP6*, *ALOX15*, *POSTN*, *CCL26*) in patients with EoE prior to PPI therapy. Individuals in this study were PPI-UR, as they demonstrated persistent esophageal symptoms and eosinophilia (≥15 eos/HPF) following the 8- to 12-week PPI trial. However, this was a heterogeneous cohort of patients with EoE who were “on-PPI” and “off-PPI” at the time of biopsy. Furthermore, there was no treatment naive assessment, and it remains unclear whether paired samples “pre- and post-PPI” were evaluated from the same individuals. The clinical assessment of PPI-R individuals with EoE before and after PPI therapy and the demonstration that the EDP esophageal transcriptome between PPI-R EoE before and after PPI therapy was similar to that observed in PPI-UR EoE suggest a similar EDP panel molecular signature between EoE endotypes (20). These studies have led to the conclusion that PPI-R and PPI-UR EoE are clinically, endoscopically, and histologically indistinguishable (13–19) and that the molecular signature is largely similar between the 2 endotypes (20, 21). However, comprehensive histologic, endoscopic, and full transcriptomic analyses of PPI-R and PPI-UR individuals with EoE, at diagnosis, in the absence of PPI exposure, and following PPI therapy are needed (30–33).

We conducted a study on treatment-naive PPI-R and PPI-UR individuals with EoE before and after PPI. We identified that (a) PPI-R and PPI-UR individuals with EoE at diagnosis have similar histologic and endoscopic disease presentations; however, transcriptomic analyses reveal common and unique DEGs in PPI-R and PPI-UR EoE at diagnosis and following PPI. (b) RNA-Seq analysis revealed dysregulation of genes associated with EoE transcriptomic signature genes, immune and inflammatory processes, mastocytosis and mast cell degranulation, and the IL-20 family of cytokines in both PPI-R and PPI-UR EoE at diagnosis. (c) Genes associated with IFN signaling and zinc ion regulation were preferentially dysregulated in PPI-R EoE, whereas pathways associated with innate immune defense, B cell and neutrophil signaling, and a sustained esophageal epithelial proliferative response were dysregulated in PPI-UR EoE. (d) PPI monotherapy induced remission in PPI-R EoE, and disease remission was associated with rectification of Th2 immune and inflammatory processes; however genes enriched in the biological processes “arachidonic acid metabolic process,” “Rap1/Ras signaling pathway,” and “neutrophil extracellular trap formation” remained dysregulated. (e) 8–12 week PPI therapy did not induce any significant reduction in histologic or endoscopic disease involvement in PPI-UR individuals with EoE; however, PPI therapy downregulated genes involved in “B cell receptor signaling pathway,” “immunoglobulin receptor binding,” “immunoglobulin complex,” and “complement activation” pathway, but not “cellular response to LPS,” “defense response to virus,” “type I IFN pathway,” and “cellular proliferation.” In silico analyses of the paired pre- and posttranscriptomics datasets identified a common set of gene drivers associated with “inflammatory response” and “defense to virus” pathways in both EoE endotypes; genes associated with “cilium movement,” “cholesterol biosynthetic pathway,” “neuropeptide signaling pathway,” and “triglyceride homeostasis” were uniquely enriched in PPI-R EoE; and genes associated with “mitotic spindle assembly,” “checkpoint chromosome segregation,” “mitotic cell cycle,” “cell division,” and “xenobiotic metabolic process” were uniquely enriched in PPI-UR EoE. These studies identify common and unique molecular signatures in PPI-R and PPI-UR EoE at diagnosis and unveil unique genes and biological processes that may drive different EoE endotypes and differential diagnosis.

Transcriptomic analyses revealed the most dysregulated genes in PPI-R and PPI-UR EoE at diagnosis were enriched for immune and inflammatory processes, including genes identified as part of the EoE transcriptomic signature (*CCL26*, *CCL24*, *TNAIP6*, *PTGDR2*, *FFAR3*, *ALOX15*, *IL13*). Furthermore, the predominant immune cell transcriptomic signature was consistent with eos (*IL5RA*, *CCL11*, *EPX*), mast cells (*CPA3, CMA1, HPGDS, HDC*, and *CTSG*), and CD4^+^ Th2 cells (*IL4*, *IL5*, *IL13*, *IL17RB*, *GATA3*) in PPI-R and PPI-UR EoE at diagnosis. Increased esophageal eos are pathognomonic for PPI-R and PPI-UR EoE, and levels correlate with esophageal remodeling and disease severity (34–36). Furthermore, previous studies have reported increased esophageal mast cell levels in EoE, and elevated levels have been associated with poor clinical responsiveness to therapy (37). scRNA-Seq analysis of human mast cell subsets in EoE has revealed the presence of a resident TPSAB1^hi^ AREG^hi^ mast cell population and two additional proinflammatory MC populations defined as KIT^hi^ IL1RL1^hi^ FCER1A^lo^ and CMA1^hi^ CTSG^hi^ in the esophagus in EoE (38). We observed increased number of esophageal tryptase^+^ mast cells and expression of mast cell genes (*CPA3*, *CMA1*, *HDC*, and *CTSG*) in both PPI-R and PPI-UR EoE at diagnosis, supporting the presence of these populations in both EoE endotypes at diagnosis. Notably, the type 2 inflammatory signature within the EoE transcriptome was amplified in PPI-UR EoE compared with PPI-R EoE at diagnosis. Moreover, the raw count values of the type 2 inflammatory genes and mast cell, eos, and CD4^+^ Th2 cell-specific gene markers were significantly higher in PPI-UR compared with PPI-R EoE at diagnosis, suggesting heightened type 2 inflammatory response in PPI-UR EoE. While we did not quantify CD4^+^ Th2 cell frequencies in biopsy samples from PPI-UR and PPI-R individuals with EoE at diagnosis, we did observe comparable levels of eos/HPF in the esophagus between the 2 EoE subtypes at diagnosis, suggesting that this signature is not reflective of differential cell numbers. Previous studies have reported comparable eos/HPF and mast cell levels between PPI-R individuals with esophageal eosinophilia and with EoE (20, 39–42).

A unique immune signature within the transcriptome that we observed in PPI-R and PPI-UR individuals with EoE at diagnosis was the presence of the IL-20 family of cytokines (*IL19*, *IL26*). A recent study reported increased mRNA expression of IL-20 subfamily of cytokines (*IL19*, *IL20*, *IL22*, *IL24*, and *IL26*) in the esophagus and serum of patients with active EoE. The active EoE group consisted of 7 of 22 individuals who underwent PPI trial, suggesting that the EoE population consisted of confirmed PPI-UR individuals with EoE and individuals with EoE with unknown PPI status (43). Our demonstration of increased *IL19* and *IL26* mRNA expression in both PPI-R and PPI-UR EoE at diagnosis indicates that this IL-20 family signature is present in both EoE endotypes. Interestingly, the IL-20 cytokines (*IL19, IL20, IL24*) have been shown to regulate expression of esophageal barrier regulatory and keratinization genes, including keratins (*KRT4*, *KRT13*, and *KRT24*), filaggrins (*FLG*, *FLG2*), and desmosome genes (*DSG1*, *DSG4*, *DSC1*, *DSC2*, *DSP*) in esophageal organoids, suggesting a role for IL-20 cytokines in esophageal epithelial barrier regulation (43). Notably, IL-20 subfamily member-mediated effects on keratinization genes and epithelial barrier integrity was dependent on STAT3- and ERK-dependent signaling (43). There are currently emerging studies revealing an important role for STAT3 in the regulation of esophageal epithelial functionality in EoE (44, 45).

The most dysregulated pathways in PPI-R EoE at diagnosis were associated with “cellular response to LPS”, “defense response to virus” (*IFIT2*, *IFIT3*, *RSAD2*), “IFN-gamma-mediated signaling” (VCAM1, *OASL*, *HLAB*, *HLAF B2M*), “type I IFN signaling pathways” (*IFI27*, *IFI6*, *IFI35*), “cellular response to zinc” (*MT1A*, *MT1B*, *MT1H*), and “xenobiotic metabolic process pathways” (*HNF4A*, *CYP2C9*, *CYP3A4*). Notably, these genes were dysregulated in PPI-UR EoE at a comparable level to PPI-R EoE (FC); however, these genes were not the most dysregulated in PPI-UR EoE. Previous studies have reported significant enrichment of genes related to IFN-α (IFNA) and IFN-γ (IFNG) responses in EoE biopsies (46). Furthermore, IFN-mediated inflammatory signaling appears to be a feature conserved between children and adults with EoE (46). Previous studies have reported that 84% T cells from biopsy samples derived from individuals with EoE express IFNG, and elevated IFNG has been detected in CD3^+^ CD8^+^ T cells from EoE biopsy tissue, suggesting CD8^+^ T cells as a primary source of IFN (47). A recent study performing scRNA-Seq profiling 421,312 individual cells from the esophageal mucosa of 7 healthy participants and 15 participants with EoE (8 active and 7 remission), identified 60 prevalent cell subsets consisting of nonhematopoietic epithelial cell and stromal and glial cell subsets and multiple hematopoietic cell subsets, including myeloid, B cell, T/natural killer lymphocytes, eos, mast cell, and erythroid (48). These analyses revealed that CD8^+^ T cells, CD4^+^ Th1 cells, and *FCERG3*^+^ NK cells were the dominant sources of *IFNG*. Furthermore, they showed that most of the 60 hematopoietic and nonhematopoietic cell subsets had elevated IFN-α and/or IFN-γ response gene signatures (48). Notably, zinc is an essential cofactor for T cell function, and zinc deficiency results in decreased T cell proliferation and altered T cell frequencies (49–51). The regulation of intracellular zinc levels involves specific transporters (ZIP and ZnT families), zinc-binding proteins like MT, and a zinc-sensing transcription factor called metal response element-binding transcription factor-1 (MTF1) (52, 53). We demonstrated that genes associated with the cellular response to zinc ions were also significantly dysregulated in PPI-R EoE. Intriguingly, PPIs are known to modulate T cell IFN production via modulating zinc transporter Zrt- and Irt-like protein 8 (Zip8) expression and distribution of intracellular zinc (54). Furthermore, zinc is known to interact with STAT3 to induce conformational changes to the STAT3 secondary structure and block STAT3-JAK2–mediated transcriptional changes in T cells (53, 55). It is interesting to speculate that PPI-induced disease remission in PPI-R EoE may in part involve blockade of zinc transporter expression and distribution of intracellular zinc. Importantly, our demonstration of the presence of the esophageal IFN signature in both PPI-R and PPI-UR EoE at diagnosis indicates that the IFN signature is independent of PPI exposure.

The unique inflammatory transcriptomic signature in PPI-UR EoE at diagnosis was associated with dysregulation of genes enriched for B cell and neutrophil signaling and an esophageal epithelial proliferative response. The observed enrichment of B cell receptor activation, downstream signal transduction, and antigen-binding activity and presentation, including dysregulation of genes, such as *IGHD*, *CD79A*, *CD19*, suggests increased B cell activity in PPI-UR EoE. There is increasing evidence of a role for IgG4 in both pediatric and adult EoE, and tissue IgG4 levels positively correlate with peak eos count and histologic involvement in pediatric EoE (53–59). Furthermore, increased IgG^+^ and IgG4^+^ cells have been linked with PPI- R EoE (56). The most notable pathways that were uniquely dysregulated in PPI-UR EoE at diagnosis were associated with proliferation. Interactive network analyses of DEGs revealed 3 major clusters associated with pathways related to cell cycle progression, cell division, and cellular proliferation and indicated an enrichment of an esophageal epithelial proliferative signal in PPI-UR EoE. We and others have previously shown that there is an increased frequency of quiescent and proliferating cells in esophageal biopsies from patients with active EoE compared with patients with EoE in remission and individuals acting as healthy controls (25, 44). We recently identified the presence of a SFRP1^+^ ESB epithelial (ESBE) cell subpopulation in EoE that expressed canonical genes consistent with the basal (*KRT14*, *KRT19*)/suprabasal (*KRT4*, *KRT6B*) epithelial cell phenotype, suggesting that this population is undergoing cellular proliferation and basolateral-to-apical differentiation and maturation (44). The *SFRP1*^+^ population uniquely expressed key canonical EoE inflammatory genes, including *CCL26*, *TNFAIP6*, *ALOX15*, and *POSTN*. Mapping the *n* = 120 DEGs unique to SFRP1^+^ ESBE cells (44) onto the PPI-UR and PPI-R EoE DEGs at diagnosis revealed a greater dysregulation of SFRP1^+^ ESBE transcriptome in PPI-UR EoE at diagnosis compared with PPI-R EoE (Supplemental Figure 5). These data further support enrichment of proproliferative phenotype in PPI-UR EoE at diagnosis. Our demonstration of a significant correlation in expression of DEGs within PPI-R and PPI-UR EoE at diagnosis with diverse datasets from previously characterized adult and pediatric EoE cohorts represents a critical validation of our findings suggesting (a) that there are conserved molecular signatures in EoE regardless of age group or PPI-R and (b) that these expression patterns represent conserved disease mechanisms across different patient populations. Finally, these studies confirm the molecular signatures identified in PPI-R and PPI-UR EoE and provide a solid foundation for understanding the mechanistic underpinnings of PPI-R and PPI-UR EoE.

A recent study by Molina-Jimenez and colleagues evaluated the protein signature of esophageal biopsies in a cohort of adult PPI responder and nonresponder patients with EoE prior to and following PPI therapy and in healthy individuals acting as controls (24). Similar to our observations, there were no significant differences in EREFS score or EoE-HSS score in responder and nonresponder individuals with EoE prior to PPI therapy. Furthermore, they demonstrated a common EoE protein signature pattern between responder and nonresponder individuals with EoE prior to PPI therapy. Notably, the authors did not identify substantial differences in esophageal mRNA levels between responder and nonresponder individuals with EoE prior to PPI therapy. The authors did identify 28 differentially expressed proteins between responder and nonresponder patients with EoE prior to PPI therapy. Mapping these *n* = 28 proteins onto our DEGs that were common (*n* = 1,889 genes) and unique (*n* = 2,195 genes unique to PPI-UR, and *n* = 407 genes unique to PPI-R) in PPI-R and PPI-UR EoE at diagnosis (Supplemental Table 10) revealed *n* = 5 genes that were common among PPI-R and PPI-UR (CFB, COL6A5, DHRS1, HK3, SLC9A3R2), *n* = 2 genes that were unique to PPI-UR (GDI1 and NLRP2), and no genes unique to PPI-R EoE. Collectively, these data suggest that these genes may be used to distinguish between responder and nonresponder EoE at the protein level; however, only GDI2 and NLRP2 were distinguishable at the mRNA level. Paired analyses of the DEGs before and after PPI in both PPI-R and PPI-UR EoE identified 1,649 genes that were PPI responsive, 647 that were PPI unresponsive, and 332 that were PPI induced. In PPI-UR EoE, 1,286 genes were PPI responsive, 2,798 were PPI resistant, and 485 genes were PPI induced. To identify common and unique gene and network drivers in PPI-R and PPI-UR EoE, we mapped the genes identified as PPI responsive in PPI-R EoE onto the PPI-unresponsive genes in PPI-UR EoE. We identified 1,140 common EoE disease gene drivers that were enriched for “inflammatory response” (*CCR3*, *IL13*, *FFAR3*, *TNFRSF4*, *PTGER2*, *HRH1*) and “defense to virus” (*STAT1*, *TLR2*, *TLR3*, *TLR8*, and *STING1*).

Wen et al. have demonstrated similar dysregulation of inflammatory response genes associated with type 2 immunity on the EDP panel between PPI-R and PPI-UR EoE (20). The common dysregulation of genes associated with defense against virus, including stimulator of IFN genes (STING) in PPI-R and PPI-UR EoE, suggests activation of host defense mechanisms (57, 58). STING is a key component of the cyclic GMP–AMP synthase/STING (cGAS/STING) signaling pathway that plays an important role in regulating cellular response to infection, cellular stress, and tissue damage (57, 58). Activation of the cGAS/STING pathway leads to downstream activation of the transcription factors, NF-κB and IFN regulatory factor 3 (IRF3), which translocate to the nucleus and induce expression of type I IFNs (57, 59). While upregulation of the STING pathway in EoE endotypes provides an explanation for the type I IFN signature, it remains to be determined whether activation of this host defense mechanism is in response to pathogen-derived or genomic or mitochondrial self-DNA. Notably, we identified 1,658 genes that were PPI-UR EoE unique gene drivers, including genes enriched for esophageal epithelial proliferation, suggesting an underlying defect in cell division, proliferation, and metabolism unique to PPI-UR EoE compared with PPI-R EoE. Interestingly, the observed inhibition of CCL26 in esophageal epithelial cells by PPIs such as omeprazole and lansoprazole is thought to involve chromatin remodeling of the *CCl26* promoter, resulting in decreased STAT6-induced *CCl26* transcriptional activity in esophageal squamous epithelial cells (31, 60). The effect of PPI appears to be specific to STAT6 as genetic variations in STAT6 have been associated with EoE and STAT6 genetic variant (rs324011) synergizes with the gain of function allele variant CYP2C19*17 to predict a PPI-UR EoE outcome (61). Recently, we identified a SFRP1^+^ inflammatory suprabasal esophageal epithelial cell (ESBE) population in EoE and herein show the presence of SFRP1^+^ ESBE cell signature in PPI-UR EoE at diagnosis and following PPI trial. Immunohistochemistry analyses demonstrated a dramatic increase SFRP1 protein within the suprabasal ESBE region and increased SFRP1^+^ cells within the epithelia in both PPI-R and PPI-UR EoE. We did not observe any significant difference in the level of staining between PPI-R and PPI-UR EoE (Supplemental Figure 6). These studies support the concept that the SFRP1^+^ ESBE cells contribute in part to the EoE phenotype and this pathway is unresponsive to PPI trial in PPI-UR individuals (44). We have previously demonstrated that induction of SFRP1 and the proproliferative phenotype in SFRP1^+^ ESBE cells was via a STAT3-dependent and STAT6- independent mechanism (44) and, thus, likely to be in part insensitive to PPI and contribute to PPI-UR.

Limitations of the current study include small sample numbers, which may be underpowered to confirm these transcriptional differences and their relationship to histologic and endoscopic characteristics. Furthermore, future validation studies are warranted, including analysis of independent pediatric EoE cohorts to confirm these transcriptional differences, longitudinal studies to validate the predictive utility of these signatures for PPI-R, and functional studies to characterize the biological pathways underlying PPI sensitivity in pediatric patients.

The identification of DEGs that distinguish between PPI-R and PPI-UR individuals at diagnosis has significant clinical implications. First, the molecular signature has the potential to serve as a diagnostic biomarker panel that could expedite clinical decision-making and guide initial therapy selection, leading to more rapid disease resolution while preventing continued disease progression and tissue remodeling (fibrostenosis) that can occur during ineffective treatment periods. Second, identification of key gene signatures and associated biological processes that are resistant to acid suppression will lead to the identification of new therapeutic targets for the treatment of PPI-UR EoE. These studies represent an important step toward precision medicine in EoE management, connecting transcriptional profiles to histological findings, and provide both mechanistic insights and practical clinical tools that could significantly improve outcomes for patients.

Herein, we performed comprehensive clinical, endoscopic and RNA-Seq analyses of PPI-R and PPI-UR EoE in treatment-naive individuals at diagnosis and identified common and divergent gene transcriptional signatures between the EoE endotypes. We identified PPI-R and PPI-UR gene programs in PPI-R and PPI-UR EoE and gene programs that are drivers of both disease endotypes. Finally, we identified that the SFRP1^+^ ESBE cell signature is present in PPI-UR EoE and is unresponsive to PPI trial. These studies show significant divergence in PPI-R and PPI-UR EoE transcriptomic signature at diagnosis and suggest that the SFRP1^+^ ESBE cell population contributes to the EoE phenotype and is unresponsive to PPI.

## Methods

### Sex as biological variable.

This study included both male and female pediatric patients. Due to the higher prevalence of EoE in males, males constituted 60% of our cohort.

### Patient cohorts.

Pediatric patients with EoE symptoms underwent EGD with biopsies at the Mount Sinai Center for Eosinophilic Disorders. EoE diagnosis required symptoms of esophageal dysfunction and ≥15 eos/HPF per consensus guidelines (23). High-dose omeprazole or lansoprazole was used to treat all patients per current guidelines for children and adults (1–2 mg/kg/d up to adult dose twice daily) (62). Based on clinical, endoscopic, and histological criteria, the treating physician was not able to predict the chance of PPI-R of these patients (14, 18, 19, 23, 63, 64). Patients were classified as PPI-R if symptoms resolved and eos counts dropped to <15/HPF or PPI-UR if criteria were not met. Individuals in the control group had 0 esophageal eos/HPF, normal endoscopy, and no esophageal or gastrointestinal pathology. Patients with concurrent gastrointestinal diseases, additional therapies, or dietary changes were excluded. Biopsies were collected in RNALater for transcriptomic analysis. EREFS and EoE-HSS scoring were performed in blinded fashion (26, 65). The study was approved by the institutional review boards at the Icahn School of Medicine at Mount Sinai and at the University of Michigan. Detailed clinical methods are provided in Supplemental Methods.

### RNA-Seq analyses.

RNA-Seq was performed on esophageal biopsies using Illumina 1.9 with quality control (RIN > 8). Raw reads were processed using FASTQC and Trimmomatic, aligned to GRCh38 using HiSAT2, and quantified with feature-counts. Duplicate sample validation showed >99% genome alignment identity and >98% sequencing depth consistency. Differential expression analysis used DESeq2 with cutoff thresholds of *P*_adj_ ≤ 0.05 and absolute log_2_FC > 1. Comparative analysis with published datasets (GSE58640, GSE197702, ref. 24) was performed using Pearson correlation and Venn diagram analyses to identify concordant gene expression patterns between PPI-R and PPI-UR groups. Detailed bioinformatics protocols are provided in Supplemental Methods.

### GO enrichment analysis.

GO biological processes were identified using DAVID Bioinformatics Resources 6.8. Common and unique genes and pathways were identified and represented as Venn diagrams (https://bioinformatics.psb.ugent.be/webtools/Venn/). Gene networks were identified by String Database, and k-mean clustering algorithm was used to identify clusters of the gene network. Figures were constructed using Inkscape 1.1.1 and Adobe Illustrator 26.0.2.

### Cell-type analysis.

Cell-specific markers were identified using literature survey and PangloDB database. Gene signatures derived from scRNA analyses of esophageal epithelial populations (GSE201153) (25) were used to identify the top 10 representative genes that distinguish esophageal epithelial subpopulations.

### Immunofluorescence staining.

FFPE distal esophageal tissue sections were processed using standard deparaffinization and antigen retrieval protocols. Following permeabilization and blocking, sections were incubated overnight with primary antibody against Ki67 (anti-Ki67 rabbit, 1:500, ab15580 Abcam) and then with secondary antibody (donkey anti-rabbit Alexa Fluor 647, 1:500, a21207 Invitrogen) and DAPI nuclear counterstain. Confocal imaging was performed using a Zeiss LSM 980 microscope with 40x objective. Detailed protocols are provided in Supplemental Methods.

### Immunohistochemistry staining.

FFPE distal esophageal tissue sections underwent standard deparaffinization, antigen retrieval, and endogenous peroxidase blocking. Following blocking with normal donkey serum, sections were incubated overnight with primary antibodies: anti-SFRP1 (rabbit, 1:100, ab126613 Abcam) and anti-Tryptase (mouse, 1:100, Dako). Detection was performed using biotinylated secondary antibodies, ABC reagents, and DAB substrate, followed by hematoxylin counterstaining. Images were acquired using a Nikon Eclipse Ti2 microscope with 40x objective. Detailed protocols are provided in Supplemental Methods.

### Cell quantification.

Ki67^+^ cells were quantified along the basement membrane using ImageJ ROI and Cell Counter plugins, with colocalization of Ki67 signal (594nm) and DAPI (405nm) required for positive identification. Results were expressed as Ki67^+^ nuclei per basement membrane unit. Tryptase^+^ mast cells were counted within defined tissue areas and reported as mast cells per area. Five patients per group (control, PPI-R, PPI-UR) were analyzed. Detailed quantification methods are provided in Supplemental Methods.

### Statistics.

We performed descriptive statistics to determine the mean and SD of the age of individuals in the control group and patients with EoE at first endoscopy. We performed 2-way ANOVA and performed Tukey’s multiple comparisons tests for histological and endoscopic parameters in individuals in the control group and patients with EoE at diagnosis and following PPI therapy. To identify the matrix of the correlation coefficients and the correlation *P* values between significant DEGs and histological parameter and Endoscopic parameter, we used flattenCorrMatrix function from Hmisc package in R. For all GO enrichment analyses using the DAVID GO analysis tool, Fisher’s Exact test was used to measure gene enrichment in different GO annotation terms, and Bonferroni, Benjamini, and FDR were calculated for each analysis for generating the adjusted *P* value. Graphs were created and statistical analyses were performed using GraphPad Prism 9.1 (GraphPad Software Incorporated). Statistical significance was represented as *P < 0.05; **P < 0.01; ***P < 0.001; ****P < 0.0001.

### Study approval.

The study was approved by the institutional review boards at the Icahn School of Medicine at Mount Sinai (10-00070) and at the University of Michigan (HUM00157078). Written informed consent was obtained from parents or legal guardians of all participants, with assent obtained from participants when developmentally appropriate as per local IRB regulations.

### Data availability.

The supporting data values file is provided in the supplemental material, and the bulk RNA-Seq dataset and corresponding R code for downstream analysis are publicly accessible through the Gene Expression Omnibus (GEO) database under accession number GSE303169.

## Author contributions

SC, AS, SM, SPH, and M Chehade designed research studies. SC, AS, SM, VG, GI, CFR, PAO, SM, M Chen, M Chehade, NG, and TMG performed research and clinical studies and generated, collected, and analyzed data. ADP performed histologic evaluations. SC, SM, PSF, M Chehade, and SPH interpreted data, performed statistical analyses, and drafted the manuscript. Co-first-authorship: SC (primary first-author) led immunofluorescence/immunohistochemistry analyses and manuscript writing. Among co–first authors, SC was listed first for immunofluorescence/immunohistochemistry analyses and writing the manuscript. AS was listed second for executed bioinformatic analyses and developed analysis pipelines. SM was listed third for computational analyses and contribution to the manuscript’s compilation.

## Supplementary Material

Supplemental data

Supplemental table 1

Supplemental table 2

Supplemental table 3

Supplemental table 4

Supplemental table 5

Supplemental table 6

Supplemental table 7

Supplemental table 8

Supplemental table 9

Supplemental table 10

Supplemental table 11

Supplemental table 12

Supporting data values

## Figures and Tables

**Table 2:**
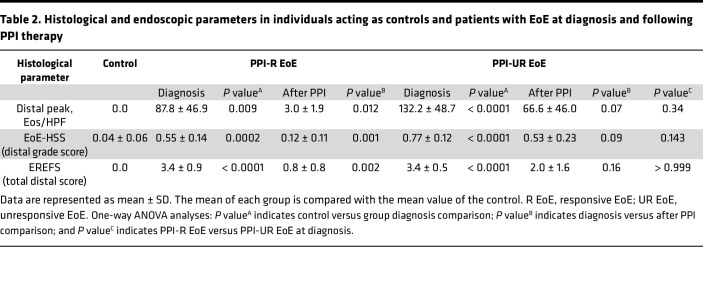
Histological and endoscopic parameters in individuals acting as controls and patients with EoE at diagnosis and following PPI therapy

**Table 1:**
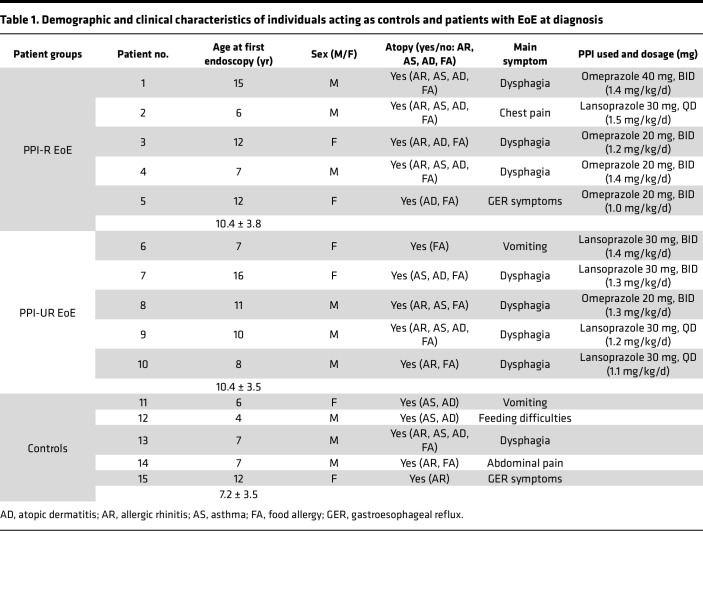
Demographic and clinical characteristics of individuals acting as controls and patients with EoE at diagnosis

## Version 1 10/08/2025

Electronic publication

## Version 2 10/17/2025

The Acknowledgments section was updated for clarity
