# Supplementary material for: Unique epithelial proliferative transcriptomic signature in proton pump inhibitor–responsive pediatric eosinophilic esophagitis
Source: JCI Insight. 2025 Oct 8;10(19):e178595. doi: 10.1172/jci.insight.178595 (PMC12513502; doi:10.1172/jci.insight.178595)
Supplement: Supplemental data [file jciinsight-10-178595-s008.pdf]

SUPPLEMENTARY FIGURE AND TABLE LEGEND

Figure S1: Comparisons of transcriptomic analysis with external data sets demonstrate significant overlap in EoE-related genes – A) Comparisons of RNAseq data with previously reported transcriptomic analysis of adult and pediatric EoE patients. **B)** Pearson correlation analysis of absolute log fold change (FC) in DEGs showing pairwise comparisons between external data sets (Molina-Jimenez PMCID: PMC11657045; Sherrill (GSE58640) and Menard Katcher (GSE197702) and PPI-R or PPI-UR individuals at diagnosis. A. Mean gene expression was obtained for each condition and represented as a log2 fold change (log2FC). Benjamini-Hochberg correction was used to adjust p-values for multiple comparisons. Genes were considered significant if they met an absolute value log2FC threshold greater than 1 and a Benjamini-Hochberg adjusted p-value (P_{adj}) less than 0.05. The Molina dataset was used as reported, with log2FC and P_{adj} values (Benjamini-Hochberg) obtained directly from the publication. B. To determine if differentially expressed genes between external datasets were concordant to DEGs, we performed pairwise comparisons between PPIR or PPIUR and each external dataset using Pearson correlation analysis. GSE197702 dataset was split into EoE and fibrostenotic subgroups for comparison.

Figure S2: Correlation analysis of histopathological parameters and biological processes specific to R-EoE and UR-EoE – (A) Comparison of distal EREF scores between control, PPI-R and PPI-UR groups at diagnosis and following PPI regimen. (**B)** Correlation analysis between total distal scores and histological parameters. (**C)** Correlation analysis between DEGs (R-EoE n = 2064 and UR-EoE, n = 3527) and total distal score at diagnosis for PPI-R (R-EoE) and PPI-UR (UR-EoE). (D) TOP 5 G) biological process pathways which correlated with EREF scores at diagnosis in PPI-R EoE at diagnosis. A. Two-way ANOVA was used to determine significance between the groups ** $p < 0.005$, *** $p < 0.0001$. Each symbol represents a different patient at diagnosis and following PPI trial. D. To identify matrix of the correlation coefficients and the correlation p-values between significant DEGs and endoscopic parameter, we used flattenCorrMatrix function from Hmisc package in R. For GO enrichment analyses using the DAVID GO analysis tool, Fisher's Exact test was used to measure gene-enrichment in different GO annotation terms, and Bonferroni, Benjamini and False Discovery Rate (FDR) are calculated for each analysis for generating the adjusted p-value.

Figure S3: The effect of PPI treatment on the transcriptome profiles between PPI-R and PPI-UR EoE – A) DEGs of associated cell types in control, EoE subjects (PPI-R and PPI-UR) ant diagnosis and following PPI. **B)** DEGs of associated cell states in control, EoE subjects (PPI-R and PPI-UR) ant diagnosis and following PPI. The gene signatures derived from scRNAseq analyses of esophageal epithelial populations (GSE201153 (25)) quiescent and proliferating basal cells, Trans1 and Trans2 suprabasal cells as well as low and high differentiated squamous epithelial cells were used to define esophageal epithelial cell population gene expression in PPI-R and PPI-UR EoE at diagnosis.

Figure S4: Common and unique transcriptome network and pathways between PPI-R and PPI-UR EoE at diagnosis – A) DEGs in common pathways of immune response and inflammatory response at diagnosis. **B)**

Effect of PPI on pathways most enriched in responsive- EoE (R-EoE). **C)** Effect of PPI on pathways most enriched in unresponsive- EoE (UR-EoE).

Figure S5: – A) Hierarchical clustering heatmap displaying the expression patterns of 120 differentially expressed genes (DEGs) that are uniquely altered in SFRP1+ ESBE cells. Expression profiles are compared across samples from healthy controls, PPI-R) patients, and non-erosive reflux disease (NERD) patients. Each column represents an individual patient sample, and each row represents a single gene. Color intensity corresponds to z-score normalized expression levels, with red indicating upregulation and blue indicating downregulation. **B)** Gene ontology analysis of these DEGs revealed enrichment in biological pathways related to innate immune signaling and Hyaluronan regulation. (B), For GO enrichment analyses using the DAVID GO analysis tool, Fisher's Exact test was used to measure gene-enrichment in different GO annotation terms, and Bonferroni, Benjamini and False Discovery Rate (FDR) are calculated for each analysis for generating the adjusted p-value.

Figure S6: SFRP1 staining in control, PPI-R and PPI-UR biopsies. A) Images of IHC-stained esophageal biopsies from control, PPI-R and PPI-UR patients showing SPRP1 localization within the tissue. EoE patients demonstrate more endogenous SFRP1 as compared to controls (inset). Top panel, X10; Middle panel inset, X20 and Bottom panel inset, X40 magnification.

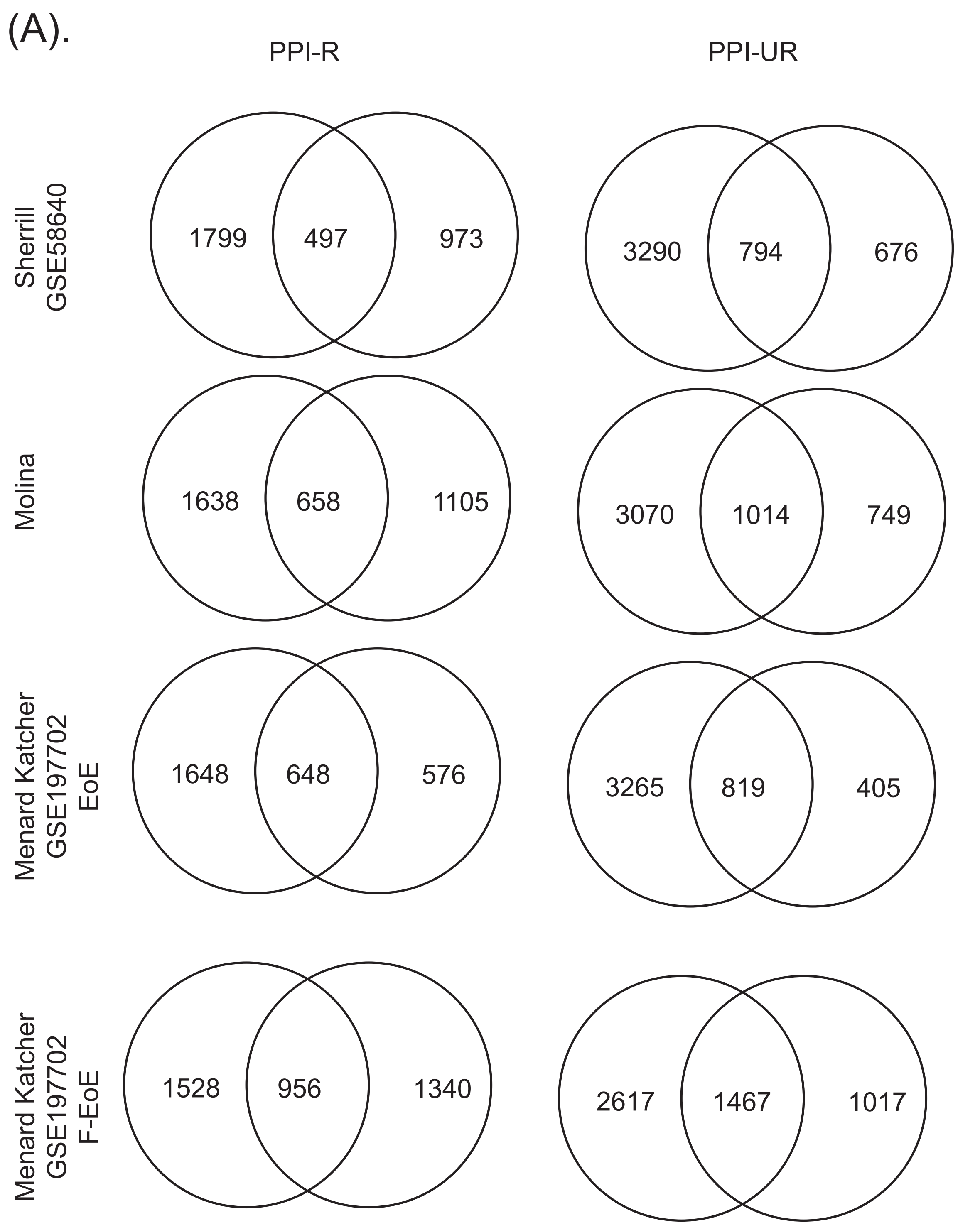

(B).

Group	R^2, p value		R^2, p value	
	**Molina-Jimenez**	**Sherrill (GSE58640)**	**Menard Katcher GSE197702 (EoE)**	**Menard Katcher GSE197702 (F-EoE)**
**PPI-R**	0.7606, $p < 3.415e^{-125}$	0.6638, $p < 1.987e^{-64}$	0.5006, $p < 1.689e^{-102}$	0.4026, $p < 1.471e^{-38}$
**PPI-UR**	0.7695, $p < 2.715e^{-199}$	0.7449, $p < 3.415e^{-141}$	0.3953, $p < 2.137e^{-86}$	0.2505, $p < 4.699e^{-24}$

Supplementary Figure S1.

(A). Endoscopic Parameter (Total Distal Score)

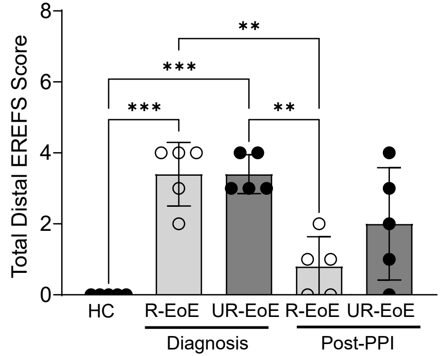

(B). Correlation analysis between Total Distal EREFS Score & Histological Parameters

Histological Parameter	Correlation value (r)	p-value
Peak Count	0.718	0.0025
Basal zone Hyperplasia	0.926	6.86E-07
Dilated Intracellular Space	0.888	9.51E-06
Total Grade Score	0.875	1.93E-05

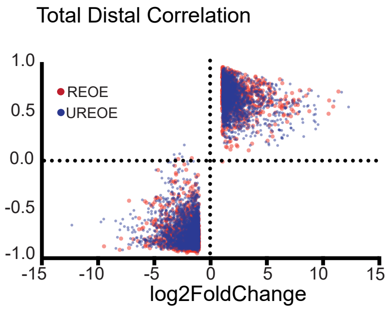

(C).

Correlation analysis with Total Distal Score at diagnosis

	R EoE DEGs (n = 2296 genes)	UR EoE DEGs (n = 4084 genes)
Total genes correlated	n = 2064 genes	n = 3527 genes

(D).

R-EoE

Biological Process	p-value	Genes
inflammatory response	2.39E-11	CD40, ECM1, CSF1, TNFAIP6, LY75
type I interferon signaling pathway	3.18E-08	IFITM3, HLA-H, RSAD2, STAT1, MX1
defense response to virus	1.91E-07	IFITM3, RTP4, CD40, UNC93B1, IFIT5
detoxification of copper ion	2.34E-07	MT2A, MT1A, MT1L, MT1M, MT1F
apoptotic signaling pathway	9.21E-07	NDUFA13, ITGAM, PTGIS, DAPL1, PDCD6

UR-EoE

Biological Process	p-value	Genes
inflammatory response	1.49E-11	IL1RN, GPR68, PARK7, LIPA, C4B
cornification	4.68E-07	FLG, SPRR2F, SPRR3, CSTA, SPRR2G
detoxification of copper ion	6.55E-07	MT2A, MT1A, MT1L, MT1M, MT1F
chromosome segregation	8.60E-07	BEX4, TOP2A, CDCA2, HJURP, TTK
mitotic spindle organization	1.06E-06	ERCC6L, ZWILCH, BUB1B, TTK, KIF11

Supplementary Figure 2

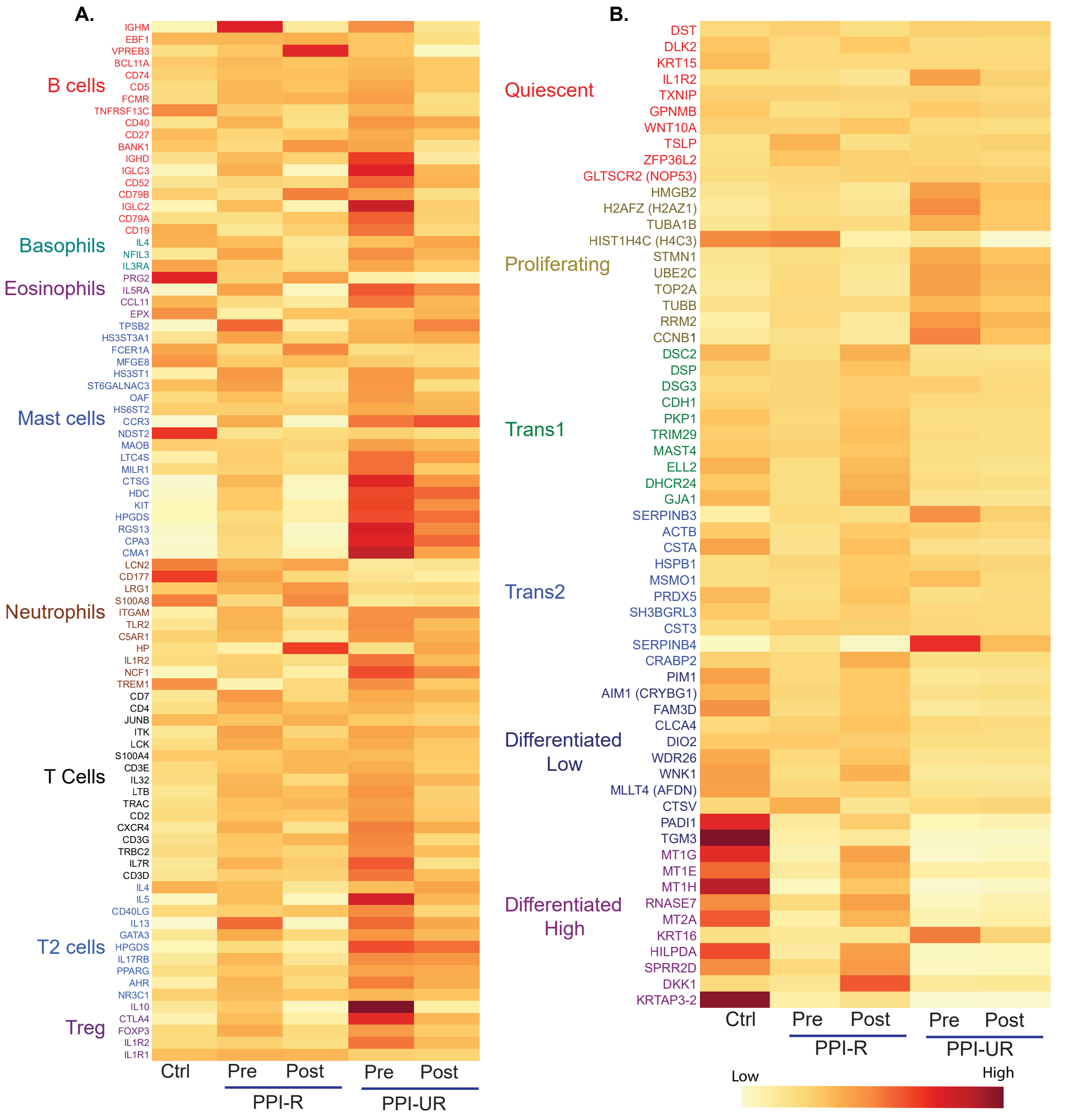

Supplementary Figure 3

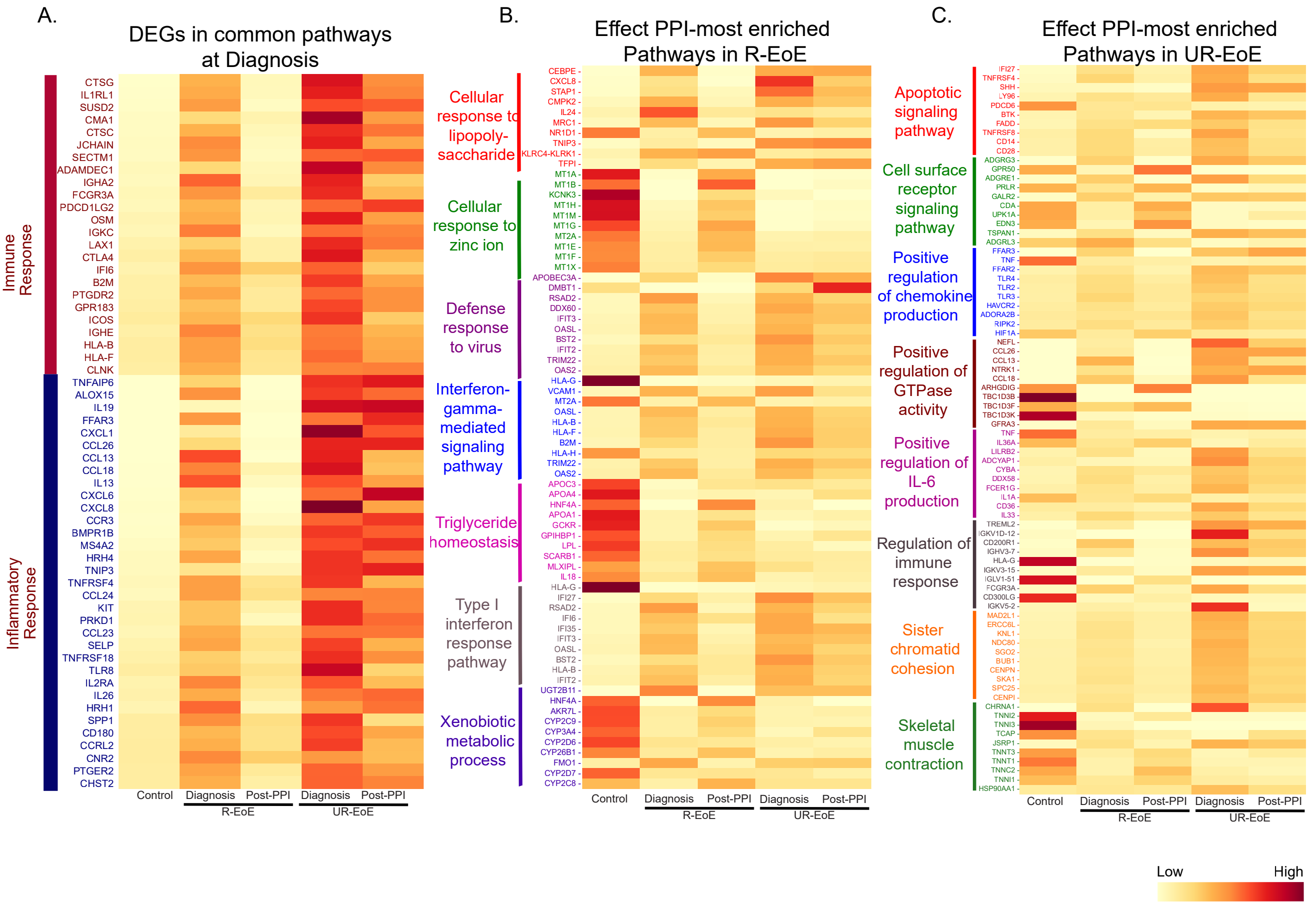

Supplementary Figure 4

A.

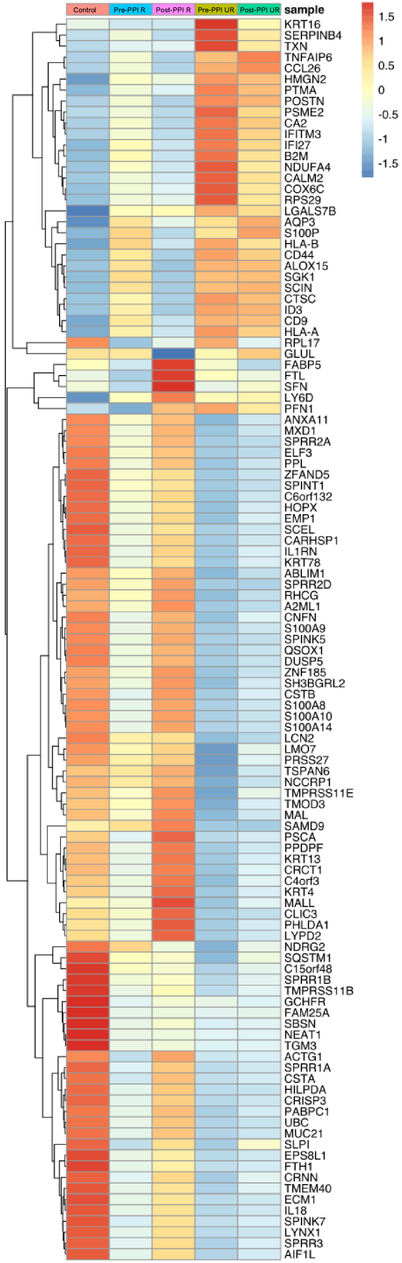

B.

Enriched Pathways	p-value
Neutrophil degranulation (R-HSA-6798695)	0.0038
Insulin-like growth factor-2 mRNA binding proteins (GF2BPs/IMPs/VICKZs) bind RNA (R-HSA_428359)	0.0049
Interleukin-18 signaling (R-HSA-90112546)	0.0056
Interleukin-1 processing (R-HSA-448706)	0.0063
Hyaluronan uptake and degradation (R-HSA-2160916)	0.0084
Hyaluronan metabolism (R-HSA-214845)	0.0111
Scavenging by class A receptors (R-HSA-3000480)	0.0132
Cell recruitment (pro-inflammatory response) (R-HSA-9664424)	0.0174
Pyroptosis (R-HSA-5620971)	0.0181
Regulation of TP53 degradation (R-HAS-6804757)	0.0249

Supplementary Figure S5

A.

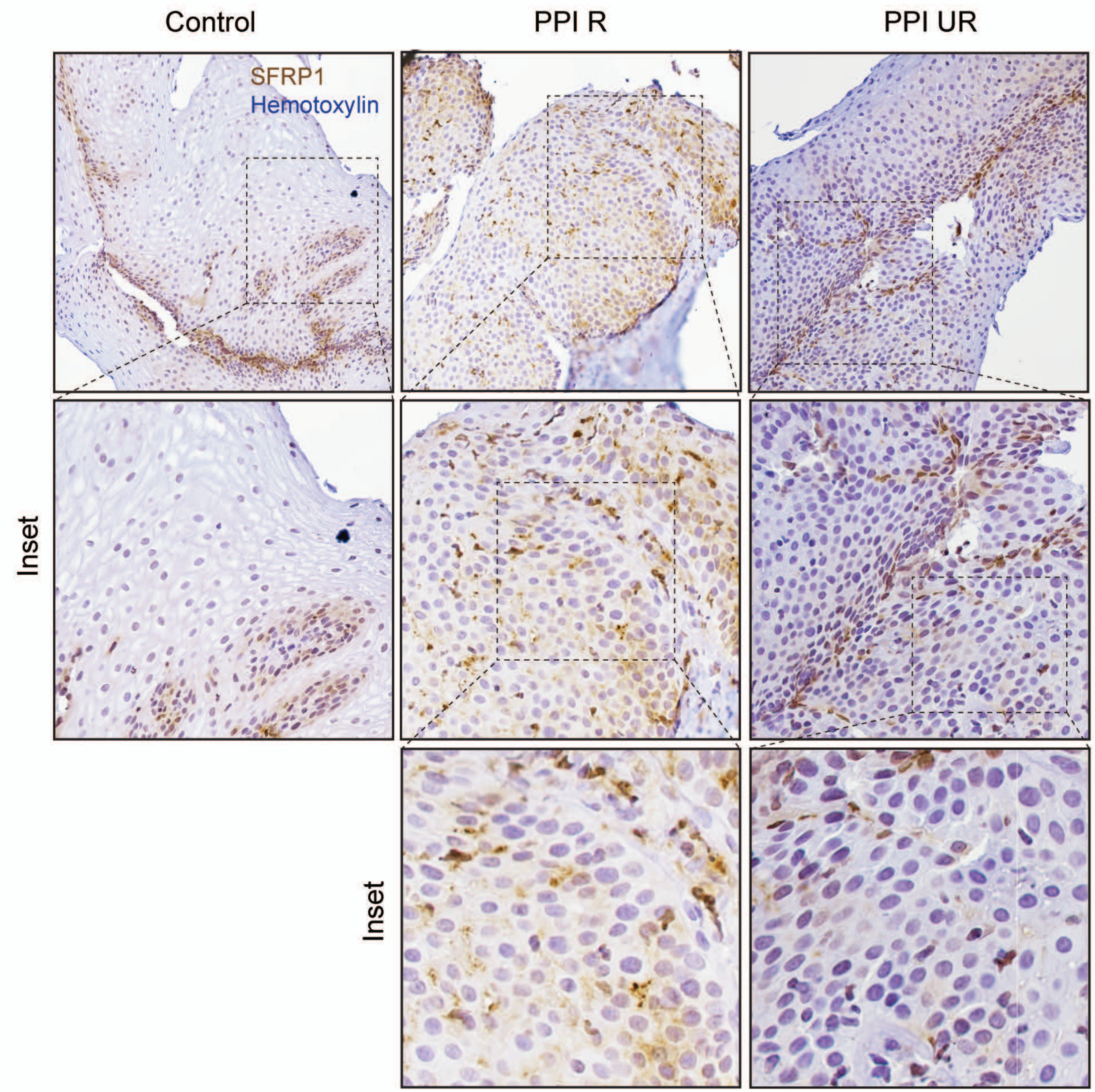

Inset

Supplementary Figure S6.

Supplementary Table S1: Endoscopic and histologic characteristics of controls and EoE patients at diagnosis and following PPI therapy.

		**EREFS Distal / proximal**		**Peak Eos /HPF Distal / proximal**		**EoEHSS distal/ proximal- grade**		**EoEHSS distal/ proximal- stage**	
**Patients**	Patient no.	Diagnosis	Post-PPI	Diagnosis	Post-PPI	Diagnosis	Post-PPI	Diagnosis	Post-PPI
**PPI-R EoE**	1	4/0	1/1	105/98	0/0	0.50/0.50	0/0.13	0.50/0.42	0/0.13
	2	4/4	2/1	72/79	3/0	0.63/0.63	0.13/0.08	0.54/0.50	0.17/0.04
	3	2/0	0/0	58/0	3/0	0.33/0.05	0.05/0	0.29/0.05	0/0
	4	3/3	1/1	161/84	5/5	0.71/0.54	0.29/0.14	0.42/0.38	0.21/0.10
	5	4/4	0/0	43/68	4/7	0.58/0.58	0.13/0.42	0.54/0.54	0.21/0.13
**PPI-UR EoE**	6	4/4	4/4	152/148	136/160	0.83/0.76	0.88/0.83	0.67/0.67	0.63/0.83
	7	4/4	3/3	66/48	36/65	0.57/0.67	0.42/0.58	0.43/0.67	0.38/0.46
	8	3/1	0/2	105/104	37/76	0.79/0.75	0.33/0.50	0.79/0.54	0.38/0.42
	9	3/4	1/1	144/155	92/115	0.88/0.83	0.63/0.71	0.79/0.79	0.63/0.67
	10	3/2	2/2	194/96	32/212	0.79/0.88	0.38/0.86	0.67/0.83	0.33/0.57
**Controls**	11	0/0		0/0		0/0		0/0	
	12	0/0		0/0		0.08/0.08		0.08/0.08	
	13	0/0		0/0		0.13/0.13		0.13/0.13	
	14	0/0		0/0		0/0		0/0	
	15	0/0		0/0		0/0		0/0	

MATERIALS AND METHODS:

Sex as biological Variable: This study included both male and female pediatric patients. Due to the higher prevalence of EoE in males, males comprised 60% of our cohort.

Patient Cohorts: Pediatric patients with symptoms suggestive of EoE and children with treatment naïve EoE being referred to the Mount Sinai Center for Eosinophilic Disorders (MSCED) for re-evaluation and follow-up care underwent an esophagogastroduodenoscopy (EGD) with biopsies. All patients had detailed collection and recording of clinical symptoms and endoscopic findings inclusive of all EoE features at the time of the endoscopy by the same clinician (M.Chehade). Control patients were identified as part of a large ongoing prospective registry at the MSCED and had provided distal esophageal biopsy for research purposes that was immediately stored in RNALater for transcriptomic analyses as per established research protocol. Note that all patients and controls had 4 distal and 4 proximal esophageal biopsies, in addition to multiple gastric antral, gastric body and duodenal biopsies, obtained during all their endoscopies by the same clinician (M.Chehade), as part of an established protocol at the MSCED. EoE was defined per recent consensus guidelines: symptoms of esophageal dysfunction and esophageal biopsies demonstrating at least 15 eosinophils per high power field (HPF) in the absence of other diseases that can cause esophageal eosinophilia (23). High dose omeprazole or lansoprazole was used to treat all patients per current guidelines for children and adults (1-2 mg/kg/day up to adult dose twice daily)(62). Based on clinical, endoscopic, and histological criteria, the treating physician was not able to predict the chance of PPI responsiveness of these patients (14, 18, 19, 23, 63, 64). Adherence to PPI was verified by the treating clinician (M.Chehade) prior to repeat endoscopy. Patients were classified as PPI-R EoE and PPI-UR EoE based on follow-up of their clinical symptoms and esophageal eosinophil counts post-therapy with PPI. Patients with symptom resolution and drop in peak esophageal eosinophil counts to < 15 eosinophils / HPF were considered PPI-responsive. Controls were defined as patients that underwent EGD with biopsies and had 0 esophageal eosinophils/HPF on histology and absent endoscopic abnormalities throughout the length of the esophagus, and no other esophageal or gastrointestinal pathology based on biopsies obtained from the gastric and duodenal mucosae. In addition, controls had no other gastrointestinal diseases diagnosed. Patients with concurrent gastrointestinal diseases such as Crohn's disease or celiac disease, concurrent evidence of gastric or duodenal eosinophilia, and patients with other disease pathology on any biopsies, both pre- and post-PPI therapy were excluded. Patients who had any additional EoE management or change in EoE management besides PPI therapy, including any dietary changes or medications, were also excluded. During all endoscopies, an additional biopsy from the distal esophagus was obtained and immediately stored in RNALater for transcriptomic analyses as per established research protocol. EREFS scoring of distal and proximal esophageal regions was performed by the same investigator (M.Chehade) in a blinded fashion as per published novel classification and grading system (65). In addition, EoE-HSS scoring was generated on distal and proximal esophageal biopsies by a pathologist (A.D.P.) in a blinded fashion per published recommendations (26), including a blind re-count of their peak esophageal eosinophil counts. The study was approved by the Institutional review boards at the Icahn School of Medicine at Mount Sinai and at the University of Michigan. All patients provided consent and assent when applicable to participate in the study.

RNAseq Analyses: RNAseq was performed on esophageal biopsies from all EoE patients at diagnosis and after PPI treatment and from controls using Illumina 1.9. RNA. Quality control was performed using Bioanalyzer RIN (RNA Integrit Number > 8). The FASTQC program and Trimmomatic tools were used to examine the quality of raw reads and filtering poor quality reads, respectively. Genome indexing was performed using Bowtie2 and the reads were aligned to the

human reference genome (GRCh38) using HiSAT2 program with the default options for paired-ends. Read counts were generated using the feature-counts function from the SourceForge Subread package. We performed multiple batch RNAseq runs of randomized duplicate samples to assess for consistency in genome alignment frequencies, depth of sequencing, and gene expression. We identified that duplicate samples had > 99% identity in Genome alignment and 98.86% identity in sequencing depth. Analyses of the gene expression (raw reads) revealed ~90.5% identity in gene expression between duplicate samples and > 99% identity in read frequency (raw read count). Informatics analyses of the individual samples (raw read counts) revealed that 100% of the observed transcripts unique to each duplicate sample was accounted for by a zero raw read count in one sample and < 7 raw read count in the duplicate sample. These low raw read counts are within the range of limit of detection of RNAseq approaches and set a threshold cut-off point of limits of detection. Our RNAseq analyses pipeline included cutoff threshold > 7 raw read count to be included in differential gene expression analyses. Downstream analysis was performed using DEseq2 in R (R Core Team, Vienna, Austria). DEGs were filtered using padj value ≤ 0.05, and absolute log2-fold-change (absLog2FC) > 1.

To validate our findings and assess the consistency of gene expression changes across studies, we performed a comparative analysis of multiple pre-existing RNA-sequencing datasets. Specifically, we compared data from proton pump inhibitor-responsive (PPI-R) and unresponsive (PPI-UR) individuals in our in-house cohort with three published datasets: GSE58640; GSE197702 and (24) including both EoE and fibrostenotic EoE subgroups. The goal was to identify shared patterns of differential gene expression and determine whether previously reported transcriptomic signatures aligned with those observed in our own dataset. Raw count matrices from the GSE58640; GSE197702 and in-house datasets were imported into R and processed using the DESeq2 package. Differential expression analysis was conducted with the design formula ~condition where each condition represented a grouping of a control sample, EoE sample, or EoE subgroup sample. Mean gene expression was obtained for each condition and represented as a log2 fold change (log2FC). Benjamini-Hochberg correction was used to adjust p-values for multiple comparisons. Genes were considered significant if they met an absolute value log2FC threshold greater than 1 and a Benjamini-Hochberg adjusted p-value (P_{adj}) less than 0.05. The Molina dataset was used as reported, with log2FC and P_{adj} values (Benjamini-Hochberg) obtained directly from the publication. To determine if differentially expressed genes between external datasets were concordant to DEGs, we performed pairwise comparisons between PPI-R or PPI-UR and each external dataset using Pearson correlation analysis. GSE197702 dataset was split into EoE and fibrostenotic subgroups for comparison. Venn diagrams were generated using the VennDiagram package in R to visualize the overlap of significant genes. Gene symbols were then used to match intersecting genes across datasets. Pearson correlation coefficients were calculated on the absolute log2FC values of intersecting differentially expressed genes to quantify similarity between comparisons. All statistical procedures and figure generation steps were performed in R using DESeq2 or R base packages. For the analyses performed at PPI-R and PPI-UR diagnosis, we compared gene expression between PPI-R and PPI-UR at diagnosis with controls. For the PPI-treatment analyses within the PPI-R or PPI-UR groups, we compared DEGs identified as described above with those identified between Control vs post-PPI within PPI-R group or Control vs post-PPI within PPI-UR group. We were unable to perform DEG analyses between Pre vs post-PPI within the PPI-R group and Pre vs post-PPI within PPI-UR group as we did not observe any DEGs that reached our pairwise comparison cut-off criteria of FDR < 0.05; log2FC > 1 in the Pre vs post-PPI within PPI-UR group.

Gene Ontology (GO) enrichment analysis: GO biological processes were identified using DAVID Bioinformatics Resources 6.8. Common and unique genes and pathways were identified and

represented as Venn diagram (https://bioinformatics.psb.ugent.be/webtools/Venn/). Gene networks were identified by String Database and k-mean clustering algorithm was used to identify clusters of the gene network. Figures were constructed using Inkscape 1.1.1 and Adobe Illustrator 26.0.2.

Cell type analysis: Cell specific markers were identified using literature survey and PangloDB database. We also utilized gene signatures derived from scRNA analyses of esophageal epithelial populations (GSE201153 (25), to identify top 10 representative genes that distinguish esophageal epithelial subpopulations. All the dysregulated genes from each comparison were mapped on the cell specific markers and heatmap was constructed for data visualization.

Immunofluorescence staining: Sections from formalin-fixed and paraffin-embedded (FFPE) distal esophageal tissues from all patients in the PPI-R, PPI-UR groups and controls were deparaffinized and rehydrated using standard histologic techniques. Antigen exposure performed at 1258C for 30 seconds in a decloaking chamber using Sodium Citrate (2.94 g/L, pH 6). Slides were then rinsed three times in 1X PBS 5 minutes each and then incubated in Blocking Buffer (PBS + 0.5% Triton X-100 + 10% normal donkey serum) (Jackson ImmunoResearch, West Grove, Pa), for 1 hour at room temperature to permeabilize and reduce nonspecific hydrophobic interaction between primary antibody and the tissue. This was followed by overnight incubation (4°C) of primary antibodies diluted in PBS + 10% normal donkey serum: anti-Ki67 (Rb) (1:500, ab15580 Abcam). The next day, slides were washed and incubated with secondary antibody, donkey anti-rabbit Alexa Fluor 647 (1:500) (a21207 Invitrogen, Thermo Fisher Scientific, Inc., Waltham, MA), at room temperature for 2 hours, following which the slides were rinsed with 1X PBS and incubated with DAPI (1:1000) for 20 mins. Slides were mounted with Vectashield Plus (Vector Labs, Newark, CA) mounting solution. Confocal images were obtained using the Zeiss LSM 980 microscope with a 40x, N.A 1.0, W Plan Apochromat objective (Zeiss) and Zeiss Zen Blue Software (v3.7). Composite images were created using ImageJ (National Institutes of Health, Bethesda, MD).

Immunohistochemistry staining: Additional FFPE distal esophageal tissue sections from the same patients were deparaffinized and rehydrated using standard histologic techniques. Tissue sections were then permeabilized in Tris-EDTA (1 mM, pH 9) with 0.1% Tween-20, and antigen exposure performed at 1258C for 30 seconds in a decloaking chamber. Slides were washed in 1X PBS three times for 5 minutes each and then incubated in 3% hydrogen peroxide (H2O2) for 15 minutes. Slides were washed again in 1X PBS three time for 5 minutes and blocked with 4% normal donkey serum (Jackson ImmunoResearch), diluted in 1X PBS, for 1 hour to reduce nonspecific hydrophobic interaction between primary antibody and the tissue. The slides were then rinsed in 1x PBS three times 5 mins each and followed by overnight incubation of primary antibody: anti-SFRP1(Rb) (1:100, ab126613 Abcam), and anti–Tryptase(M) (1:100, Dako) at 4°C. The next day, samples were washed 3 times with 1x PBS for 5 minutes each and incubated with biotinylated antibodies (anti–rabbit IgG & anti-mouse IgG) secondary antibody for 60 minutes. Slides were rinsed in 1x PBS thrice and incubated with ABC reagents (Vectastain; Vector Labs, Burlingame, Calif) for 30 minutes at room temperature. 3,3'-Diaminobenzidine (DAB) peroxidase substrate was prepared immediately before use (Vectastain; Vector Labs), applied to the slides, and observed for brown staining. Slides were added to tap water to stop the reaction and counterstained with Harris Modified Hematoxylin Solution for two dips (Sigma-Aldrich). Slides were then dehydrated using standard histologic techniques and mounted with Cytoseal mounting media (Thermo Fisher Scientific, Kalamazoo, Mich). Stained slides were imaged using Nikon Eclipse Ti2 with a 40x, N.A 0.95 Plan Apochromat λD (Nikon), using the Nikon Elements software (AR 6.10) and analyzed using ImageJ (National Institutes of Health, Bethesda, MD).

Cell Quantification: Ki67-positive cell quantification was performed using the ROI and Cell Counter plugins in ImageJ (National Institutes of Health, Bethesda, MD). Basement membrane was identified and traced in the captured confocal images. Using the Cell Counter plugin, Ki67-positive cells were counted using the 594nm channel as the source. A cell present along the basement membrane was considered Ki67-positive only when the signal in the 594nm channel colocalized with the 405nm channel (DAPI) and was negative in the 488nm (autofluorescence). Total Basement membrane lengths and number of Ki67-positive cells were summarized for each image and reported as “Ki67-positive nuclei/ Basement Membrane unit” per patient. 5 patients per group (Control, PPI-R and PPI-UR) were analyzed. Tryptase-positive cell quantification was performed using the ROI, Measurements and Cell Counter plugins in ImageJ (National Institutes of Health, Bethesda, MD). Tissues were outlined using ROI manager and their area was calculated using the Measurements plugin. Using the Cell Counter plugin, we counted the number of cells positive for Tryptase staining and granular in appearance. Total tissue area and number of Tryptase-positive cells were summarized for each image and reported as “Mast Cells/ Area” per patient. 5 patients per group (Control, PPI-R and PPI-UR) were analyzed.

Statistics: We performed descriptive statistics to determine the mean and standard deviation of the age of control and EoE patients at first endoscopy. We performed Two-way ANOVAs and performed Tukey’s multiple comparisons tests for histological and endoscopic parameters in control and EoE patients at diagnosis and following PPI therapy. To identify matrix of the correlation coefficients and the correlation p-values between significant DEGs and histological parameter and Endoscopic parameter, we used flattenCorrMatrix function from Hmisc package in R. For all GO enrichment analyses using the DAVID GO analysis tool, Fisher's Exact test is used to measure gene-enrichment in different GO annotation terms, and Bonferroni, Benjamini and False Discovery Rate (FDR) are calculated for each analysis for generating the adjusted p-value. Graphs and statistical analyses were performed using GraphPad Prism 9.1 (GraphPad Software Incorporated, La Jolla, CA, USA). Statistical significance was represented as * : $p<0.05$; ** : $p<0.01$; *** : $p<0.001$; **** : $p<0.0001$.

Study approval: The study was approved by the Institutional review boards at the Icahn School of Medicine at Mount Sinai (10-00070) and at the University of Michigan (HUM00157078). All patients provided written informed consent and assent when applicable to participate in the study.

Data availability: The supporting data values file is provided as supplementary material, while the bulk RNA sequencing dataset and corresponding R code for downstream analysis are publicly accessible through the Gene Expression Omnibus (GEO) database under accession number GSE303169.
