# Supplementary material for: Unique epithelial proliferative transcriptomic signature in proton pump inhibitor–responsive pediatric eosinophilic esophagitis
Source: JCI Insight. 2025 Oct 8;10(19):e178595. doi: 10.1172/jci.insight.178595 (PMC12513502; doi:10.1172/jci.insight.178595)
Supplement: Supplemental table 1 [file jciinsight-10-178595-s009.pdf]

Supplementary Table S1: Endoscopic and histologic characteristics of controls and EoE patients at diagnosis and following PPI therapy.

		**EREFS Distal / proximal**		**Peak Eos /HPF Distal / proximal**		**EoEHSS distal/ proximal- grade**		**EoEHSS distal/ proximal- stage**	
**Patients**	Patient no.	Diagnosis	Post-PPI	Diagnosis	Post-PPI	Diagnosis	Post-PPI	Diagnosis	Post-PPI
**PPI-R EoE**	1	4/0	1/1	105/98	0/0	0.50/0.50	0/0.13	0.50/0.42	0/0.13
	2	4/4	2/1	72/79	3/0	0.63/0.63	0.13/0.08	0.54/0.50	0.17/0.04
	3	2/0	0/0	58/0	3/0	0.33/0.05	0.05/0	0.29/0.05	0/0
	4	3/3	1/1	161/84	5/5	0.71/0.54	0.29/0.14	0.42/0.38	0.21/0.10
	5	4/4	0/0	43/68	4/7	0.58/0.58	0.13/0.42	0.54/0.54	0.21/0.13
**PPI-UR EoE**	6	4/4	4/4	152/148	136/160	0.83/0.76	0.88/0.83	0.67/0.67	0.63/0.83
	7	4/4	3/3	66/48	36/65	0.57/0.67	0.42/0.58	0.43/0.67	0.38/0.46
	8	3/1	0/2	105/104	37/76	0.79/0.75	0.33/0.50	0.79/0.54	0.38/0.42
	9	3/4	1/1	144/155	92/115	0.88/0.83	0.63/0.71	0.79/0.79	0.63/0.67
	10	3/2	2/2	194/96	32/212	0.79/0.88	0.38/0.86	0.67/0.83	0.33/0.57
**Controls**	11	0/0		0/0		0/0		0/0	
	12	0/0		0/0		0.08/0.08		0.08/0.08	
	13	0/0		0/0		0.13/0.13		0.13/0.13	
	14	0/0		0/0		0/0		0/0	
	15	0/0		0/0		0/0		0/0	
